# Self-perceived life course sleep duration trajectories and risk and age at onset of Parkinson’s disease

**DOI:** 10.1038/s41531-025-01202-w

**Published:** 2025-12-14

**Authors:** Yi Fang, Rebecca Hardy, Kristine Yaffe, Simon J. Little, Caroline M. Tanner, Yue Leng

**Affiliations:** 1 Department of Psychiatry and Behavioral Sciences, University of California, San Francisco, CA USA; 2 School of Sport, Exercise, and Health Sciences, Loughborough University, Loughborough, UK; 3 Department of Epidemiology & Biostatistics and Department of Neurology, University of California, San Francisco, CA USA; 4 Movement Disorders and Neuromodulation Center, Department of Neurology, Weill Institute for Neurosciences, University of California, San Francisco, CA USA

**Keywords:** Parkinson's disease, Epidemiology

## Abstract

Sleep disturbances are both prodromes and potential risk factors for Parkinson’s disease (PD), yet associations between long-term sleep patterns and PD remain unclear. We analyzed data from two online cohorts—PPMI-Online (*n* = 15,905) and Fox Insight (*n* = 1929)—to examine associations between self-reported sleep duration trajectories across life stages and PD risk and age at onset (AAO). Latent class growth analysis identified distinct trajectories, linear and logistic regression examined their associations with PD risk and AAO, adjusting for demographics, lifestyle, and comorbidities. In PPMI-Online, sleep duration was generally stable in early adulthood with divergence in midlife. Compared to stable sleepers (7–8 h/day), individuals with midlife reductions (6–7 to ≤5–6 h/day: OR = 1.90; 7–8 to ≤6–7 h/day: OR = 1.64) and persistent short sleep (≤6 h/day: OR = 1.41) had higher PD risk, independent of comorbidities. Similar patterns were observed in probable prodromal PD. Persistent short sleep and sleep decline were also linked to earlier AAO (up to –4.23 years) across cohorts. These findings suggest that midlife sleep reduction may signal early PD, while chronic short sleep may represent a modifiable risk factor, highlighting the need for prospective studies to explore early detection and prevention potential.

## Introduction

Sleep disturbances are common nonmotor symptoms of Parkinson’s disease (PD), affecting 60% to 90% of patients^1,2^. Evidence indicates a bidirectional relationship between PD and altered sleep, even before symptomatic onset^3,4^. Sleep deprivation exacerbates synucleinopathy in rodent models^5^, and poor sleep in human patients is associated with faster motor progression^6^, indicating the role of sleep disturbances in PD pathogenesis. Furthermore, PD pathology disrupts the sleep/wake neural circuitry before overt clinical motor symptoms^3^. This disruption is exemplified by rapid eye movement sleep behavioral disorder (RBD), a well-established prodromal marker of PD^7^.

Population-based studies report higher rates of insomnia or hypersomnia^8–10^, and variations in sleep duration^11,12^ preceding PD diagnosis. However, these studies typically focus on isolated time points, primarily the decade before diagnosis, overlooking potential life course changes in sleep duration^13^. Sleep trajectories have been associated with adverse neurological outcomes, such as dementia^14–16^, but no study has examined the association between life course sleep duration and PD. Given that prodromal PD can span decades^17^ and early-life sleep patterns may contribute to neurodegeneration later in life^18^, it is crucial to investigate early-life sleep trajectories and their connection to PD onset. Such research is critical for disentangling the role of sleep disturbances as early or risk factors for PD, offering opportunities for early detection and intervention.

In this study, we identified self-reported sleep duration trajectories from adolesence to late adulthood using data from the Parkinson’s Progression Marker Initiative Online (PPMI-Online) and Fox Insight (FI). We assessed their relationship with PD risk and age at onset (AAO) in PPMI-Online, validating AAO findings in FI. We hypothesized that midlife sleep changes could serve as early markers of PD and that sleep durations below recommendations (<7 h/night^19,20^) in early adulthood or midlife would be associated with higher risk and earlier AAO of PD. We further examined whether these patterns were also associated with probable prodromal PD, as defined by the MDS research criteria^21^. Sensitivity analyses excluded participants with self-reported RBD, anxiety or depression, dementia, and examined sex as a potential effect modifier.

## Results

### Study participants

**Fig. 1:**
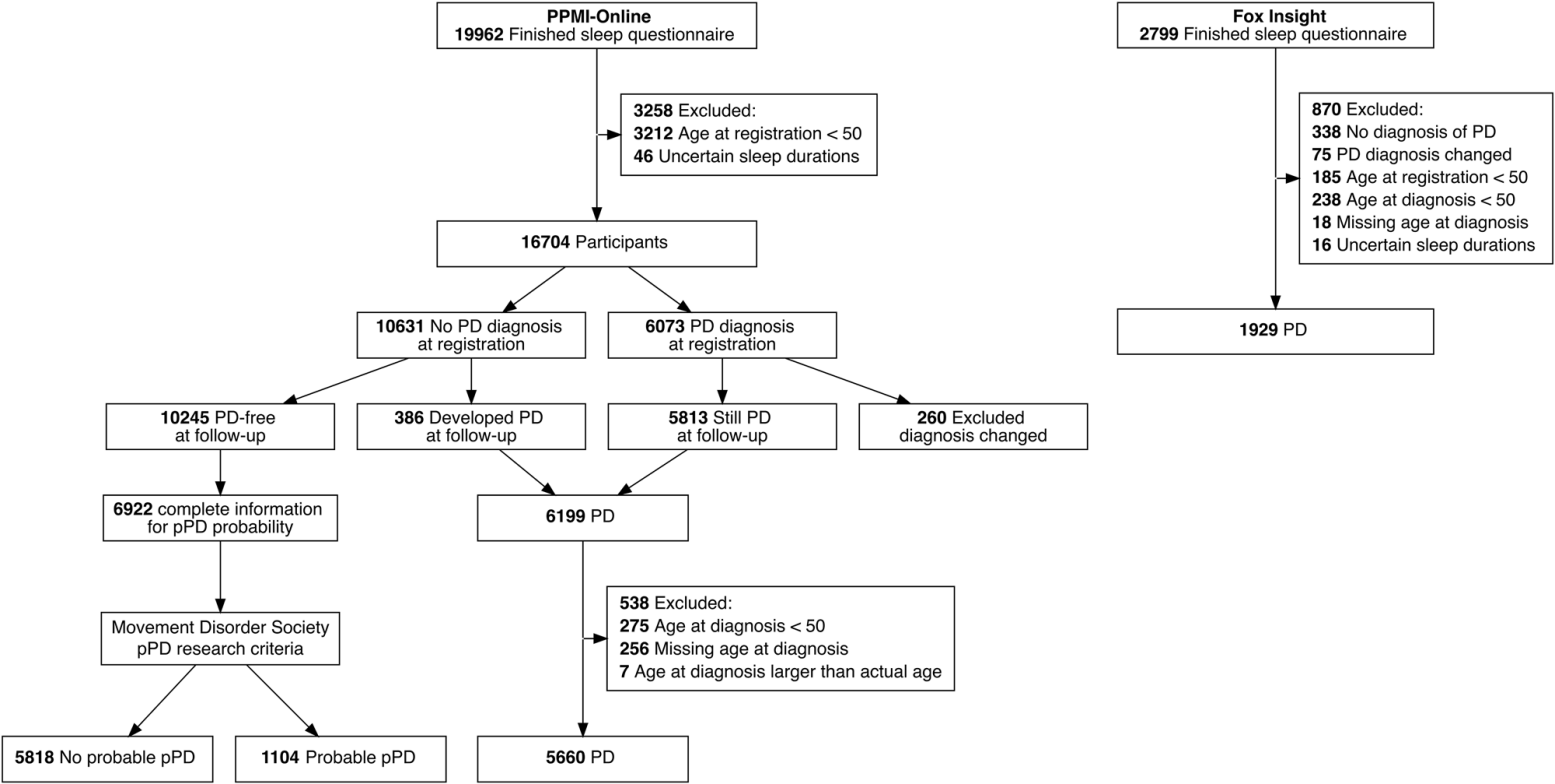
Flow diagram of study participants. PD Parkinson’s disease, pPD prodromal Parkinson’s disease.

A flowchart of study participants is presented in Fig. 1. The final sample in the discovery cohort included 11,728 participants from PPMI-Online (5660 with PD and 10,245 without PD). Among the latter, 6922 had complete data and were classified as “probable prodromal PD (pPD)” (*n* = 1104) or “no probable pPD” (*n* = 5818) based on the Movement Disorders Society criteria^21^ (80% probability cutoff^22^). The validation FI sample included 1929 participants with PD; the 338 participants without PD were excluded due to limited sample size.

For the current analysis, we applied identical exclusion criteria to both cohorts, including age at registration <50 years, young-onset PD (AAO < 50 years^23^), diagnosis changed during follow-up, missing age at diagnosis, reported age at diagnosis older than the participant’s current age, and all sleep duration responses marked as “prefer not to answer” or “don’t know”.

Demographic and clinical characteristics of participants is summarized in Table 1: 15,905 from PPMI-Online [mean age at sleep report: 67.2 ± 7.94 years; 8795 (55.3%) female; mean follow-up: 22.7 ± 8.71 months] and 1929 from FI [mean age at sleep report: 67.5 ± 6.87 years; 940 (48.7%) female; mean follow-up: 68.1 ± 10.1 months]. PD mean AAO was 65.3 ± 7.57 in PPMI-Online and 62.9 ± 6.96 in FI. In PPMI-Online, participants without PD were younger, more likely female, and had higher education, income, and positive PD family history compared to those with PD. Across subgroups (“no probable pPD,” “probable pPD,” and “PD”; Supplmentary Table 1), motor and non-motor symptoms showed a clear progression, supporting the validity of the pPD criteria.

**Table 1:** Demographic and clinical characteristics of the study participants

	PPMI-Online			Fox Insight
	No PD	PD	*P* value ^a^	PD
	(*n* = 10,245)	(*n* = 5660)		(*n* = 1929)
Age at sleep report (years), Mean (SD)	65.8 (7.97)	69.7 (7.27)	<0.001	67.5 (6.87)
Age at onset of PD (years), Mean (SD)	NA	65.3 (7.57)	NA	62.9 (6.96)
Follow-up since enrollment (months), Mean (SD)	23.0 (8.58)	22.2 (8.91)	<0.001	68.1 (10.1)
Sex (Female), No (%)	6428 (62.7%)	2367 (41.8%)	<0.001	940 (48.7%)
Race (Non-White), No (%)	399 (3.9%)	196 (3.6%)	0.12	38 (2.0%)
Education ^b^	17.0 (3.20)	16.5 (3.56)	<0.001	68.3% > = Bachelor’s degree
Yearly Household Income, No (%)			<0.001	
<$50,000	1029 (10.0%)	707 (12.5%)		452 (23.4%)
$50,000 to $99,999	2637 (25.7%)	1517 (26.8%)		625 (32.4%)
More than $100,000	4971 (48.5%)	2473 (43.7%)		562 (29.1%)
Prefer not to answer	1331 (13.0%)	791 (14.0%)		287 (14.9%)
Missing	277 (2.70%)	172 (3.0%)		3 (0.2%)
Family history of PD No (%)	5965 (58.2%)	1664 (29.4%)	<0.001	525 (27.2%)
RBD1Q			<0.001	
No	7566 (73.9%)	2646 (46.7%)		1141 (59.1%)
Yes	2320 (22.6%)	2766 (48.9%)		779 (40.3%)
Not sure or Prefer not to answer	358 (3.5%)	245 (4.4%)		NA
MDS-UPDRS part I, Mean (SD)	5.26 (3.95)	8.84 (4.51)	<0.001	NA
MDS-UPDRS part II, Mean (SD)	1.77 (3.49)	10.5 (7.49)	<0.001	10.8 (7.45)
PDAQ-15, Mean (SD)	54.6 (6.10)	51.0 (9.11)	<0.001	51.8 (8.98)
GDS-15, Mean (SD)	2.41 (2.90)	3.68 (3.44)	<0.001	3.46 (3.37)
PAS, Mean (SD)	7.23 (6.74)	9.44 (7.55)	<0.001	NA
ESS, Mean (SD)	5.95 (3.61)	7.18 (4.20)	<0.001	NA
PDSS2, Mean (SD)	11.7 (6.89)	16.5 (8.46)	<0.001	NA

*PD* Parkinson’s disease, *RBD1Q* Rapid Eye Movement Sleep Behavior Disorder Single-Question Screen, *MDS-UPDRS* Movement Disorder Society-sponsored revision of the Unified Parkinson’s Disease Rating Scale, *PDAQ15* The Penn Parkinson’s Daily Activities Questionnaire-15, *GDS-15* Geriatric Depression Scale-15, *PAS* Parkinson Anxiety Scale, *ESS* Epworth Sleepiness Scale, *PDSS2* The Parkinson’s Disease Sleep Scale–2.

^a^Independent t-test for continuous variable, chi-square test for categorical variable.

^b^Numeric (years of education) for PPMI-Online, and categorical for Fox Insight.

### Sleep duration trajectories (PPMI-Online)

**Fig. 2:**
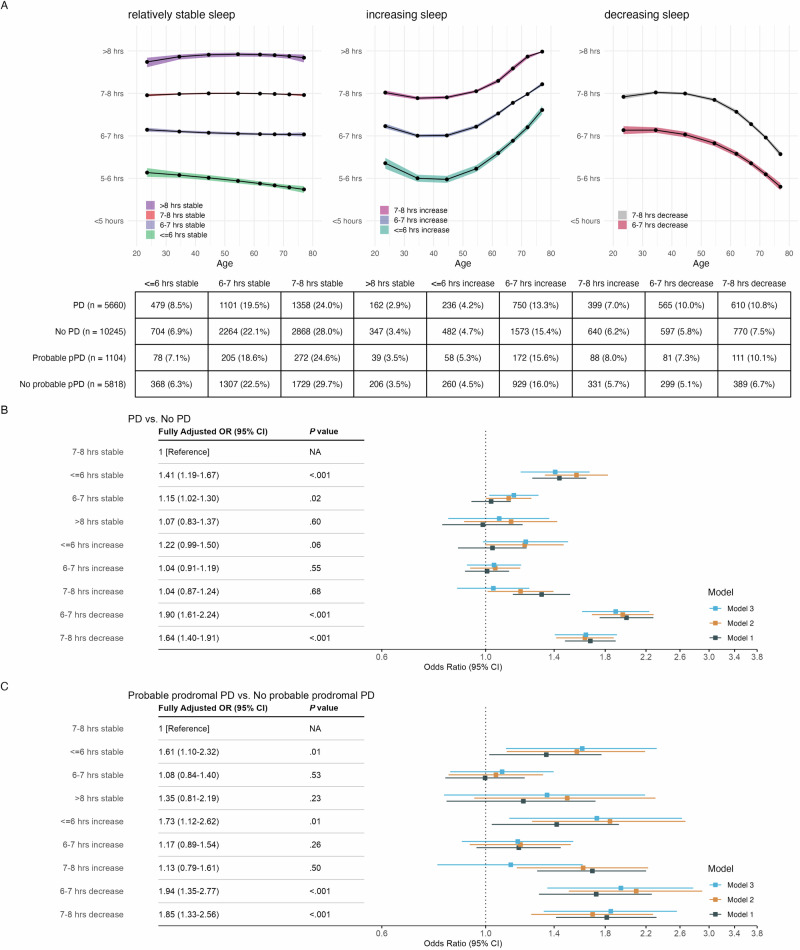
Sleep duration trajectories identified from PPMI-Online and associations with Parkinson’s disease risk. PD Parkinson’s disease, pPD prodromal Parkinson’s disease. Panel **A**: Sleep duration trajectory groups identified through latent class growth analysis, shown in separate panels based on trends emerging after age 50. Dots and shaded areas represent mean trajectories with 95% confidence intervals. The accompanying table shows the count and proportion of participants for each trajectory pattern. Panel **B**: Logistic regression results for the association between sleep duration trajectory patterns and risk of PD, using participants without a PD diagnosis as the reference group. Panel **C**: Logistic regression of sleep duration trajectory patterns and risk of “Probable pPD”, with the “No probable pPD” group as the reference. Model 1 was unadjusted. Model 2 adjusted for age at sleep report, sex, race, family history of PD, education, and income. Model 3 further adjusted for history of anxiety, depression, brain injury, diabetes, hypertension; Rapid Eye Movement Sleep Behavior Disorder Single-Question Screen at the time of sleep report; lifetime caffeine intake, smoking status, and lifetime physical inactivity.

PPMI-Online sleep duration trajectories (Fig. 2A) remained relatively stable from the 20s to the 40s, with distinct trends emerging after the 50s (depicted in separate panels). Groups were labeled according to both baseline sleep in early adulthood (with categories: >8 h, 7–8 h, 6–7 h, ≤6 h) and changes after age 50 (categorized as: stable, decrease, or increase). These trajectory shapes were largely consistent in sensitivity analyses. The “≤6 h stable” group had a lower age at sleep report, smaller percentage of White individuals, fewer years of education, and lower household income. Groups with changes after 50s (“7–8 h increase”, “6–7 h decrease”, “7–8 h decrease”) had higher age at sleep report and lower income. The “>8 h stable” group included more females (Supplementary Table 2).

### Sleep duration trajectories and PD risk (PPMI-Online)

Compared to the “7–8 h stable” group, those with consistently shorter sleep (“≤6 h stable”: OR = 1.41, 95% CI 1.19–1.67, *P* < 0.001, all ORs from Model 3) or decreasing sleep after age 50 (“6–7 h decrease”: OR = 1.90, 95% CI 1.61–2.24, *P* < 0.001, “7–8 h decrease”: OR = 1.64, 95% CI 1.40–1.91, *P* < 0.001) showed increased PD risk. The associations remained consistent across all adjustments (Fig. 2B). The “6–7 h stable” group also showed a modestly higher risk of PD after adjustment (OR = 1.15, 95% CI 1.02–1.30, *P* = 0.02).

For probable pPD, consistently shorter sleep (“≤6 h stable”: OR = 1.61, 95% CI 1.10–2.32, *P* = 0.01) and decreasing sleep after 50 (“6–7 h decrease”: OR = 1.94, 95% CI 1.35–2.77, *P* < 0.001; “7–8 h decrease”: OR = 1.85, 95% CI 1.33–2.56, *P* < 0.001) were similarly associated with higher risks, consistent with the findings for PD risk. In addition, the “≤6 h increase” group also exhibited elevated pPD risk (OR = 1.73, 95% CI 1.12–2.62, *P* = 0.01).

### Sleep duration trajectories and PD AAO (PPMI-Online)

**Fig. 3:**
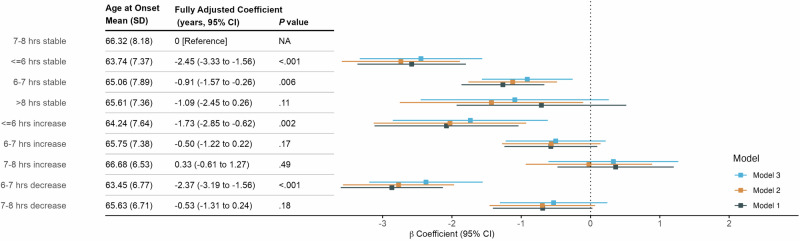
Association of life course sleep duration with age at onset of Parkinson’s disease in PPMI-Online. Model 1 was unadjusted. Model 2 adjusted for sex, race, family history of PD, education, and income. Model 3 further adjusted for history of anxiety, depression, brain injury, diabetes, hypertension; Rapid Eye Movement Sleep Behavior Disorder Single-Question Screen at the time of sleep report; lifetime caffeine intake, smoking status, and lifetime physical inactivity.

Compared to the “7–8 h stable” group, short sleep in early adulthood or decreasing sleep in midlife was associated with earlier PD AAO (Fig. 3). After full adjustment, the largest effect size was observed in the “≤6 h stable” group (*β* = −2.45 years, 95% CI −3.33 to −1.56, *P* = < 0.001) and “6–7 h decrease” group (*β* = −2.37 years, 95% CI −3.19 to −1.56, *P* = < 0.001), followed by the “≤6 h increase” (*β* = −1.73 years, 95% CI −2.85 to −0.62, *P* = 0.002) and “6–7 h stable” (*β* = −0.91 years, 95% CI −1.57 to −0.26, *P* = 0.006).

### Sleep duration trajectories and associations with PD AAO (FI)

**Fig. 4:**
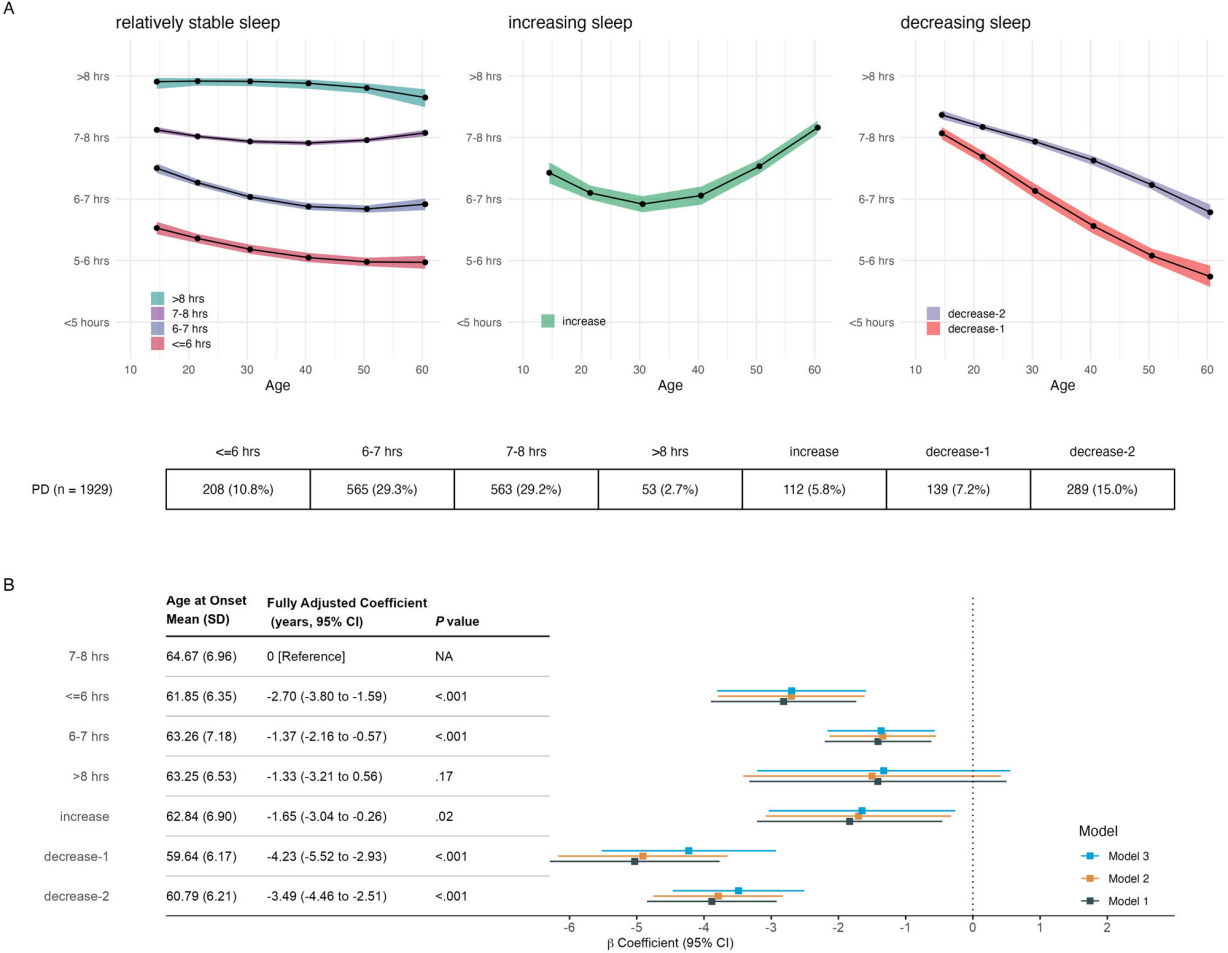
Sleep duration trajectories identified from Fox Insight and associations with Parkinson’s disease age at onset. Panel **A**: Dots and shaded areas represent mean trajectories for each class with 95% confidence intervals. The table displays the count and proportion of each trajectory pattern. Panel **B**: Sleep duration trajectory patterns and age at onset of PD. Model 1 was unadjusted. Model 2 adjusted for sex, race, family history of PD, education, and income. Model 3 further adjusted for history of anxiety, depression, brain injury, diabetes, hypertension; Rapid Eye Movement Sleep Behavior Disorder Single-Question Screen at the time of sleep report; lifetime caffeine intake, smoking status, and lifetime physical inactivity.

Most FI trajectories (Fig. 4A) exhibited moderate sleep decline from adolescence to adulthood. Among them, four groups (labeled “>8 h”, “7–8 h”, “6–7 h”, “≤6 h”) maintained relatively stable during midlife. Two groups showed continuous declines: “decrease-1,” with decreases earlier in life, and “decrease-2,” with declines more evident during midlife. The “increase” group exhibited a sleep increase after the 40s, with early adulthood sleep duration between 6–7 h, resembling the “6–7 h increase” group in PPMI-Online. Groups with short sleep (“≤6 h”, “6–7 h”) and decreasing sleep (“decrease-1”, “decrease-2”) had lower education levels. The decrease groups also had a younger age at sleep report (Supplmentary Table 3).

Compared to the “7–8 h” group, groups with less sleep exhibited earlier AAO (Fig. 4B). The effect size was most pronounced in the decrease groups (“decrease-1”: *β* = −4.23 years, 95% CI −5.52 to −2.93, *P* < 0.001; “decrease-2” (*β* = −3.49 years, 95% CI −4.46 to −2.51, *P* < 0.001), followed by “≤6 h” (*β* = −2.70 years, 95% CI −3.80 to −1.59, *P* < 0.001), “increase” (*β* = −1.65 years, 95% CI −3.04 to −0.26, *P* = 0.02), and “6–7 h” (*β* = −1.37 years, 95% CI −2.16 to −0.57, *P* < 0.001).

### Sensitivity analyses

The associations remained consistent across sexes and were robust among participants without RBD symptoms or diagnoses throughout follow-up, those without a history of anxiety or depression, and after excluding individuals with possible dementia (Supplementary Figs. 1–7). Additionally, shorter baseline sleep duration and decreasing sleep during midlife were independently linked to an increased PD risk and earlierer AAO (Supplementary Fig. 8).

## Discussion

In two large, PD-centered online cohorts, we identified distinct self-reported life course sleep duration trajectories and examined their associations with PD risk and AAO. PPMI-Online results highlighted midlife, particularly around age 50, as a critical turning point in sleep duration. Declining sleep during midlife was associated with up to a 90% higher risk of PD and pPD. Chronic short sleep from early adulthood was associated with a 1.4-fold increased PD risk. Additionally, both PPMI-Online and FI cohorts indicated that shorter-than-recommended sleep in early adulthood or midlife was associated with an earlier AAO, with such patterns associated with earlier AAO by up to 2.5 to 4.2 years.

PPMI-Online and FI trajectories exhibited both shared and distinct patterns, likely reflecting differences in life stage coverage and participant demographics. Around 50% of PPMI-Online participants were in trajectory groups showing changes after age 50, and 60% of FI participants belonged to groups showing declines from adolescence to the 30s. These findings align with previously identified turning points in sleep duration at ages 33 and 53^13^. However, stable groups might be overestimated due to categorical presentation, potentially missing subtle changes.

Midlife (ages 40–60) is increasingly recognized as a critical period marked by accelerated declines in gene expressions, brain connectivity, and cognitive function^24,25^. We observed that decreasing sleep after age 50 was associated with increased PD and pPD risk, suggesting that declining sleep is evident in prodromal PD and continues into clinical PD. This association remained robust even among participants without self-reported RBD, anxiety, or depression, indicating that decreasing sleep may be an early manifestation of PD itself, rather than a consequence of common prediagnostic comorbidities observed in a subset of patients^26^. Conversely, the “≤6 h increase” group showed an increased risk of pPD but not PD, suggesting that increasing sleep during midlife, particularly after chronic short sleep, may indicate prodromal PD. However, our study lacked an ultra-long sleep group (>8 h/day), limiting comparisons to previous reports linking ≥9 h/day with higher PD risk^27,28^.

Given the long prodromal window, it is challenging to determine the direction of the relationship between sleep disturbances and PD. Our study indicates that short sleep in early adulthood or midlife is associated with increased risk and earlier AAO of PD, potentially preceding the onset of PD by up to six decades. These findings suggest that chronic short sleep might be a risk factor, with prolonged short sleep likely preceding neurodegeneration. Our results also underscore the public health relevance of addressing insufficient sleep, particularly in minoritized populations^29^, and its adverse effects on aging-related outcomes^30^. As primary or secondary preventive trials for PD are being planned^31^, promoting healthy sleep hygiene may represent a scalable strategy for reducing risk or delaying onset.

Our findings align with mechanistic evidence from animal and human studies. Sleep disturbances may arise from early PD pathology affecting the locus coeruleus^32^, hypothalamus^33^, and dopaminergic systems^34^. Conversely, sleep deprivation exacerbates synucleinopathy^5^, possibly through mitochondrial and oxidative stress^35^, neuroinflammation^36^, impaired glymphatic clearance^37^, and synaptic homeostasis^38^. Notably, the locus coeruleus is particularly vulnerable to insufficient sleep and may contribute to PD pathogenesis^39,40^. Furthermore, chronic sleep loss in young adult rodents has long-lasting neurological effects^41^, and is associated with neurodegeneration later in life^18^, supporting our findings that short sleep in early adulthood might contribute to PD in later years.

This study offers a novel life course perspective by leveraging two large, PD-centered online cohorts with a large participant pool and sleep duration data spanning adolescence to age 80. However, several limitations should be considered. First, the retrospective self-reported sleep duration may introduce recall bias, although evidence suggests such information can be accurate^42,43^. In this study, participants could select “don’t know” for uncertain responses, and results remained robust after excluding possible dementia. Additionally, the sleep trajectory turning points aligned with a previous large-scale study^13^, enhancing the credibility of self-reported sleep. While self-reported sleep duration modestly correlates with objectively measured sleep^44^, subjective pereptions may still capture clinically relevant perceptual aspects of sleep disruption^45,46^. A related concern is that recall may differ by diagnostic status, potentially biasing associations. Notably, similar patterns were seen in participants with prodromal PD, even without a PD diagnosis, supporting a link to underlying disease rather than recall bias. Second, pPD was classified solely using self-reported markers, without confirmation from clinician-based evaluations or objective biomarkers such as DAT-SPECT or synuclein-based assays. This limitation may increase the risk of both false-positive and false-negative classifications. However, the observed stepwise progression of motor and non-motor scores across the “no probable pPD”, “probable pPD”, and “PD” groups supports the internal validity of the pPD classification in our study. Furthermore, the study population was predominantly White, with high socioeconomic status and education, and participants without PD were enriched for PD risk factors. Further studies are needed to assess generalizability. Finally, while the findings suggest short sleep as a potential PD risk factor, the observational nature of this study limits causal inferences. Future research should also consider the potential of major events like the COVID-19 pandemic, which may have affected sleep patterns differently across patients and age groups.

In this large-scale study involving individuals with and without PD, we found that the 50s represent a significant turning point in sleep duration. Monitoring self-perceived sleep duration changes, particularly after age 50, is crucial, as declining sleep duration at this stage may signal a higher risk and earlier onset of PD. Additionally, chronic short sleep was associated with an increased risk and earlier AAO of PD, suggesting short sleep since early adulthood as a potential risk factor and intervention targets. These findings support a life course framework for understanding the role of sleep in PD pathogenesis and underscore the need for future prospective studies to validate these associations and examine other sleep dimensions for early detection and prevention strategies across the life span.

## Methods

### PPMI-online and FI study design

PPMI-Online and FI are ongoing online longitudinal observational cohorts involving participants with and without PD. Convenience sampling was used to recruit participants aged 18 and older with minimal eligibility criteria. Recruitment was conducted through online channels, such as social media and email outreach, and on-site methods, including clinician referral and PD-related research events. Recruitment began in 2017 for FI^47,48^ and 2021 for PPMI-Online^49^. Both cohorts rely on repeated web-based assessments of PD status, symptoms, comorbidities, and lifestyle factors, thereby broadening the representativeness of PD research beyond clinic-based samples. Participants report PD status and symptoms quarterly via an online platform and complete occasional questionnaires, including those on sleep duration. The sleep questionnaire was administered during the 3rd and 15th months for PPMI-Online and between October 2017 and March 2019 for the FI study. For participants with multiple sleep reports, the most recent data were used for analysis. Study protocols were approved by institutional review boards (FI: New England IRB# 120160179; PPMI-Online: WCG IRB# 20211908), and all participants provided online informed consent. This study was conducted in accordance with the Declaration of Helsinki.

### Self-perceived sleep duration across the lifespan

Participants reported their typical sleep duration during current and prior age periods. Response options were categorical and included: less than 5 h, 5–6 h, 6–7 h, 7–8 h, more than 8 h, don’t know, and prefer not to answer. Responses of “don’t know” or “prefer not to answer” were treated as missing values.The PPMI-Online study captured data spanning adulthood, with more granular age categories during late adulthood (18–29, 30–39, 40–49, 50–59, 60–64, 65–69, 70–74, 75–79, and 80 or older). The FI study focused on earlier life stages (12–17, 18–25, 26–35, 36–45, 46–55, 56–65, and 66 or older), capturing transitions from adolescence to adulthood.

### Outcome measures

PD status was based on self-reported physician diagnosis at registration and follow-up, with previous studies confirming the reliability of self-reported PD in an online setting^50^. The self-reported age at first PD diagnosis was used as a proxy for PD AAO, as these measures were highly correlated^51^.

### Motor and non-motor symptom measures

Several standardized instruments were collected to characterize the clinical profile of study participants, and their descriptive results are summarized in Table 1. The Movement Disorder Society–Unified Parkinson’s Disease Rating Scale (MDS-UPDRS) parts I and II evaluate non-motor and motor aspects of daily living, respectively^52^. The Penn Parkinson’s Daily Activities Questionnaire–15 (PDAQ-15) measures daily cognitive functioning in PD^53^. The Geriatric Depression Scale–15 (GDS-15)^54^ and the Parkinson Anxiety Scale (PAS)^55^ assess depressive and anxiety symptoms. The Epworth Sleepiness Scale (ESS)^56^ evaluates excessive daytime sleepiness, while the Parkinson’s Disease Sleep Scale–2 (PDSS-2)^57^ captures PD-specific sleep disturbances. The RBD Single-Question Screen (RBD1Q)^58^ was used to assess dream enactment behavior suggestive of REM sleep behavior disorder. Higher scores indicate greater symptom burden for all instruments, except for the PDAQ-15, where higher scores reflect worse cognition.

For the present analysis, we used the assessment conducted at the same visit as the sleep duration questionnaire (or the closest available visit if not collected at the same time) to align symptom measures with the timing of the sleep report.

### Statistical analysis

We used latent class growth analysis (LCGA) to classify individuals into distinct sleep duration trajectory groups separately for PPMI-Online and FI, incorporating both linear and quadratic time components. LCGA identifies latent classes with similar trajectory patterns over time, assuming homogeneity within each class^59^. In the initial phase, we identified trajectory groups in participants with complete sleep duration data from ages 18–29 to 75–79 for PPMI-Online (*n* = 2643) and ages 12–17 to 56–65 for FI (*n* = 1668), with the midpoint of each age range approximating age. We excluded the “80 or older” and “66 or older” groups due to limited sample sizes and broad age ranges. Participants lacking sleep data for all required periods were excluded to avoid overrepresentation of younger groups and sparse data for older groups. Those who selected “prefer not to answer” or “not sure” for any age period were also excluded to avoid potential bias from recall errors. In the next phase, all participants with at least one valid data point were assigned to the trajectory class with the highest membership probability. To assess trajectory consistency and robustness, we visually compared trajectory groups from (1) participants with complete data across age periods (main analysis) versus all participants, (2) those with PD versus without PD, and (3) those with RBD symptoms/diagnoses versus those without RBD. We tested models with 1–9 classes, selecting the optimal model based on lower Bayesian Information Criteria (BIC) and interpretability^60^. BIC decreased as the number of classes increased from 1 to 9 for both PPMI-Online and FI. We selected the 9-class models for PPMI-Online and the 7-class model for FI, as additional classes showed similar patterns with no meaningful differences and limited sample size. Further LCGA details are provided in *Supplementary*.

We analyzed associations between sleep duration trajectories and PD risk using logistic regression, and PD AAO using linear regression. Given possible non-monotonic changes during the prodromal phase^61,62^, we performed seperate binomial logistic regressions for “PD” (with “No PD” as the refrence) and “probable pPD” (with “no probable pPD” as the reference). We built three models: Model 1 (unadjusted), Model 2 (adjusted for age at sleep report [for risk analyses only], sex, race, family history of PD, education, and income), and Model 3 (further adjusted for self-reported lifetime history of anxiety, depression, brain injury, diabetes, hypertension; RBD Single-Question Screen^63^ at the time of sleep report; lifetime caffeine intake, smoking status, and lifetime physical inactivity). We did not include age at sleep report as a covariate for AAO as AAO typically precedes the sleep report and is minimally influenced by the age at sleep report. Covariates, selected based on their known or suspected associations with sleep and PD^64^, were self-reported, with the closest report to sleep report used for repeated measures. All models met assumptions, with no variance inflation factor exceeding 5, indicating minimal multicollinearity.

Sensitivity analyses included: (1) restricting to participants without self-reported RBD (*n* = 8395), given its association with shorter sleep^65^, (2) restricting to participants without history of anxiety or depression (*n* = 8566), given the divergent effects of these conditions and their treatments on sleep, (3) excluding participants with possible dementia (Penn Parkinson’s Daily Activities Questionnaire-15 < 43^53^) to enhance recall accuracy (*n* = 14,405), and (4) exploring sex as a possible effect modifier. Furthermore, as baseline sleep during early adulthood and trends during midlife are critical components of the sleep duration trajectories, we also investigated whether these two factors are independently associated with PD risk and AAO.

We performed data analysis from January to December 2024 using R (version 4.3.2). Major packages included “lcmm”^66^ (version 2.1.0), “stats” (version 4.3.2), “ggplot2” (version 3.4.4). All tests were 2-sided, with statistical significance set at *P* < 0.05. This study followed the Strengthening the Reporting of Observational Studies in Epidemiology (STROBE) reporting guideline.

## Supplementary information

Supplementary materials

## Data Availability

Final data used in this article were obtained from the PPMI study (tier1 data downloaded on 03 July 2024, [https://www.ppmi-info.org/access-data-specimens/download-data](https:/www.ppmi-info.org/access-data-specimens/download-data), RRID:SCR\_006431) and the FI study (version: December 2024 archived monthly data, [https://foxinsight-info.michaeljfox.org/insight/explore/insight.jsp](https:/foxinsight-info.michaeljfox.org/insight/explore/insight.jsp)). For up-todate information on the PPMI study, visit [www.ppmi-info.org](http:/www.ppmi-info.org). For up-to-date information on the FI study, visit [https://foxinsightinfo.michaeljfox.org/insight/explore/insight.jsp](https:/foxinsightinfo.michaeljfox.org/insight/explore/insight.jsp).
