# Supplementary material for: Self-perceived life course sleep duration trajectories and risk and age at onset of Parkinson’s disease
Source: NPJ Parkinsons Dis. 2025 Dec 14;11:357. doi: 10.1038/s41531-025-01202-w (PMC12738539; doi:10.1038/s41531-025-01202-w)
Supplement: Supplementary file 1 — Supplementary materials [file 41531_2025_1202_MOESM1_ESM.pdf]

Contents of Supplementary Materials

Supplementary Material 1. Calculation of Probability of Prodromal Parkinson's Disease in PPMI-Online Participants without Parkinson's Disease Using the MDS Research Criteria (**Page 2-6**)

Supplementary Material 2. Latent Class Growth Analysis of Life Course Sleep Durations (**Page 7-23**)

Supplementary Figure 1. PPMI-Online Sensitivity Analysis – Possible RBD excluded (**Page 24**)

Supplementary Figure 2. PPMI-Online Sensitivity Analysis – Depression, anxiety excluded (**Page 25**)

Supplementary Figure 3. PPMI-Online Sensitivity Analysis – Possible dementia excluded (**Page 26**)

Supplementary Figure 4. PPMI-Online Sensitivity Analysis – Sex stratified (**Page 27, 28**)

Supplementary Figure 5. Fox Insight Sensitivity Analysis – Depression, anxiety excluded (**Page 29**)

Supplementary Figure 6. Fox Insight Sensitivity Analysis – Possible dementia excluded (**Page 29**)

Supplementary Figure 7. Fox Insight Sensitivity Analysis – Sex stratified (**Page 30**)

Supplementary Figure 8. Baseline Sleep During Early Adulthood, Midlife Changes of Sleep and PD Risk and Age at Onset in PPMI-Online (**Page 31, 32**)

Supplementary Table 1. Demographic and Clinical Characteristics of Subgroups in PPMI-Online (**Page 33**)

Supplementary Table 2. Demographic Information of Sleep Trajectory Patterns of PD Participants in the PPMI-Online Study (**Page 34**)

Supplementary Table 3. Demographic Information of Sleep Trajectory Patterns of PD Participants in the Fox Insight Study (**Page 35**)

Supplementary Material 1. Calculation of Probability of Prodromal Parkinson's Disease in PPMI-Online Participants without Parkinson's Disease Using the MDS Research Criteria

Prodromal PD (pPD) is a stage characterized by the presence of non-motor symptoms or subclinical parkinsonian symptoms, occurring before the onset of classic motor symptoms definitive of PD. In the PPMI-Online study, among 10,245 participants without PD, 6,922 had complete information on the risk and prodromal markers of prodromal PD necessary for calculating the probability of pPD. Participants who responded with "don't know" or "prefer not to answer" to one or more items were excluded. The probability of pPD was calculated based on the Movement Disorder Society research criteria,[1] with a cut-off of 80% probability[2] used to determine "probable pPD". Consequently, 1,104 participants were classified as having "probable pPD", while 5,818 were categorized as "No probable pPD". This latter group was considered to have a low risk of developing PD in the future, given the high negative predictive value of the criteria.[3]

Probability = age-dependent prior probability of Parkinson's disease * likelihood ratio of risk or prodromal markers

Prior probabilities of Parkinson's disease based on age[2]

Age	Prior Probability (%)
50-54	0.4
55-59	0.75
60-64	1.25
65-69	2.0
70-74	2.5
75-79	3.5
>=80	4.0

Likelihood Ratios of Risk and Prodromal Markers According to the 2019 MDS Research Criteria for Prodromal Parkinson's Disease[1]

		LR +	LR -	Variables and descriptions	Derived variable
Risk markers	Male sex	1.2 (male)	0.8 (female)	SEX_OL	
	Regular pesticide exposure	1.5	NA	WRKPEST_EXPOSLIFETM_OL "Over your lifetime, have you ever had a JOB in which you mixed, applied, or were exposed in some other way to any type of pesticide, including herbicides (kill weeds), fungicides (kill fungus/mold), insecticides (kill insects), or fumigants (gas used to kill fungus/mold or insects)?"	
	Occupational solvent exposure	1.5	NA	WRKCHEMEXP_100DYSOLV_NONE_OL In lifetime, did not use solvents or degresers 100 days or more at work	
	Nonuse of caffeine	1.35	0.88	CAFFEINE_COFFEE_REG_IN_LIFE_OL Ever regularly drank caffeinated coffee (at least once per week for 6 months or longer) CAFFEINE_BLACK_REG_IN_LIFE_OL	CAFFEINE_COFFEE_REG_IN_LIFE_OL==0&CAFFEINE_BLACK_REG_IN_LIFE_OL==0&CAFFEINE_GREEN_REG_IN_LIFE_OL==0&CAFFEINE_REGSODA_REG

	LR +	LR -	Variables and descriptions	Derived variable
			Ever regularly drank caffeinated black tea (at least once per week for 6 months or longer)<br>CAFFEINE_GREEN_REG_IN_LIFE_OL<br>Ever regularly drank caffeinated green tea (at least once per week for 6 months or longer)<br>CAFFEINE_REGSODA_REG_IN_LIFE_OL<br>Ever regularly drank caffeinated regular soda (at least once per week for 6 months or longer)<br>CAFFEINE_DIETSODA_REG_IN_LIFE_OL<br>Ever regularly drank caffeinated diet soda (at least once per week for 6 months or longer)<br>CAFFEINE_ENERGY_REG_IN_LIFE_OL<br>Ever regularly drank caffeinated energy drinks or products (at least once per week for 6 months or longer)	_IN_LIFE_OL==0&CAFFEINE_DIETSODA_REG_IN_LIFE_OL==0&CAFFEINE_ENERGY_REG_IN_LIFE_OL==0
Nonsmoking				
Current smoker	NA	0.51	SMOKE_CIG_CURR_OL<br>Currently regularly smoking cigarettes<br>SMOKE_REG_IN_LIFE_OL<br>Smoked regularly (at least 1 cigarette per day for 6 months or longer) in lifetime<br>FIVE_PACKS_IN_LIFE_OL<br>Smoked 100 or more cigarettes (5 packs) in lifetime	SMOKE_CIG_CURR_OL == 1
Never smoker	1.2	NA		FIVE_PACKS_IN_LIFE_OL == 0
Former smoker	NA	0.91		SMOKE_CIG_CURR_OL == 0 & SMOKE_REG_IN_LIFE_OL == 1
First-degree relative with PD	2.5	NA	BIOMOMPD_OL<br>Has a Biological Mother with PD or Parkinsonism<br>BIODADPD_OL<br>Has a Biological Father with PD or Parkinsonism<br>HAS_FULSISPD_OL<br>Has a Full Sister with PD or Parkinsonism<br>HAS_FULBROPD_OL<br>Has a Full Brother with PD or Parkinsonism<br>HAS_KIDSPD_OL<br>Has a Child with PD or Parkinsonism	BIOMOMPD_OL==1|BIODADPD_OL==1|HAS_FULSISPD_OL==1|HAS_FULBROPD_OL==1|HAS_KIDSPD_OL==1
or<br>Known gene mutation (with intermediate-strength penetrance)	LR+ dependent on age-related penetrance	NA	Not Available	

		LR +	LR -	Variables and descriptions	Derived variable
	or Polygenic risk score (PRS)	1.57 (highest quartile of PRS scores)	0.45 (lowest quartile)	Not Available	
	SN hyperechogenicity	3.4	0.38	Not Available	
	Diabetes mellitus (type II)	1.5	0.97	DIABETES_DIAGNOSED_OL<br>Diabetes diagnosis	
	Physical inactivity	1.3	0.91	PHYSACT_AGE1829_VIGRS_PHYSACT_OL … PHYSACT_AGE80OVR_VIGRS_PHYSACT_OL<br>From ages 18-29/30-39/40-49/50-59/60-64/65-69/70-74/75-79/age 80 and up, hours/week of vigorous physical activity<br>1: Not at all<br>2: Less than 1 hour<br>3: 1-4 hours<br>4: 5-10 hours<br>5: More than 10 hours/week<br>PHYSACT_AGE1829_MODRT_PHYSACT_OL … PHYSACT_AGE80OVR_MODRT_PHYSACT_OL<br>From ages 18-29/30-39/40-49/50-59/60-64/65-69/70-74/75-79/age 80 and up, hours/week of moderate physical activity<br>1: Not at all<br>2: Less than 1 hour<br>3: 1-4 hours<br>4: 5-10 hours<br>5: More than 10 hours/week	Reported “not at all” or “less than 1 hour per week” of moderate and vigorous activity at all life stages
	Low plasma urate levels	1.8 (in men)	0.88 (in men)	Not Available	
Prodromal markers	PSG-proven RBD	130	0.65	RBD_PSG_CONF_OL<br>Sleep laboratory (PSG) confirmation of RBD	
	Possible RBD (questionnaire)	2.8	0.89	RBD1Q_ACT_DREAMS_OL<br>Been told or suspect you act out your dreams	
	Dopaminergic PET/SPECT	43.3	0.66	Not Available	

<table>
<tr><th></th><th></th><th>LR +</th><th>LR -</th><th>Variables and descriptions</th><th>Derived variable</th></tr>
<tr><td></td><td>clearly
abnormal</td><td></td><td></td><td></td><td></td></tr>
<tr><td></td><td>Subthreshold
parkinsonism</td><td>9.6</td><td>0.55</td><td>Not Available</td><td></td></tr>
<tr><td></td><td>or
Abnormal
quantitative
motor testing</td><td>3.5</td><td>0.60</td><td>Not available</td><td></td></tr>
<tr><td></td><td>Olfactory loss</td><td>6.4</td><td>0.40</td><td>HYPOSMIA_OL
Do you have any problems with your sense of smell?</td><td></td></tr>
<tr><td></td><td>Constipation</td><td>2.5</td><td>0.82</td><td>NUM_BOWEL_MVMNTS_OL
What is your usual number of bowel movements per day?
1: Less than once every other day
2: Once every other day
3: Once per day
4: Two per day
5: Three per day
6: More than three per day
7: Unknown
8: Prefer not to answer
FREQ_USE_LXTVS_OL
How often do you typically use laxatives to help you move your bowels?
0: Never
1: Daily
2: 2 to 3 times per week
3: Weekly
4: Once per month
5: Every 2 months
6: Rarely
7: Not sure
8: Prefer not to answer</td><td>Constipation:
NUM_BOWEL_MVMNTS_OL == 1 |
FREQ_USE_LXTVS_OL == 1 |
FREQ_USE_LXTVS_OL == 2 |
FREQ_USE_LXTVS_OL == 3
No constipation:
NUM_BOWEL_MVMNTS_OL %in% c(2,3,4,5,6) &
FREQ_USE_LXTVS_OL %in% c(0, 4, 5, 6)</td></tr>
<tr><td></td><td>Excessive
daytime
somnolence</td><td>2.7</td><td>0.86</td><td>Epworth Sleepiness Scale</td><td>Epworth Sleepiness Scale total score > 10[4]</td></tr>
<tr><td></td><td>Orthostatic
hypotension</td><td>18.5</td><td>0.88</td><td>Not available</td><td></td></tr>
</table>

	LR +	LR -	Variables and descriptions	Derived variable
(OH) – neurogenic OH				
Symptomatic OH	3.2	0.80	NP1LTHD_OL (UPDRS item 1.12)	NP1LTHD_OL >= 1
Erectile dysfunction	3.4 (in men)	0.87 (in men)	ED_DIAGNOSIS_OL	
Urinary dysfunction	2.0	0.90	NP1URIN_OL (UPDRS item 1.10)	NP1URIN_OL >= 1
Depression (± anxiety)	1.6	0.88	DEPRESSION_DIAGNOSED_OL	
Global cognitive deficit	1.8	0.88	The Penn Parkinson's Daily Activities Questionnaire-15	PDAQ-15 total score < 43[5]

References

1. Heinzel S, Berg D, Gasser T, et al. Update of the MDS research criteria for prodromal Parkinson’s disease. *Movement Disorders*. 2019;34(10):1464-1470. doi:10.1002/mds.27802
2. Berg D, Postuma RB, Adler CH, et al. MDS research criteria for prodromal Parkinson’s disease. *Movement Disorders*. 2015;30(12):1600-1611. doi:10.1002/mds.26431
3. Kulcsarova K, Skorvanek M. Challenges and Future of the International Parkinson and Movement Disorder Society Prodromal Parkinson’s Disease Criteria: Are We On the Right Track? *Movement Disorders*. 2024;39(4):637-643. doi:10.1002/mds.29724
4. Johns M, Hocking B. Daytime Sleepiness and Sleep Habits of Australian Workers. *Sleep*. 1997;20(10):844-847. doi:10.1093/sleep/20.10.844
5. Brennan L, Siderowf A, Rubright JD, et al. The Penn Parkinson’s Daily Activities Questionnaire-15: Psychometric properties of a brief assessment of cognitive instrumental activities of daily living in Parkinson’s disease. *Parkinsonism & Related Disorders*. 2016;25:21-26. doi:10.1016/j.parkreldis.2016.02.020

Supplementary Material 2. Latent Class Growth Analysis of Sleep Durations

We applied latent class growth analysis (LCGA) to classify individuals into distinct groups of sleep duration trajectories, using the "lcmm" package (version 2.1.0) in R. The ordinal, 5-class Likert-type sleep duration responses were handled using a "thresholds" link function.[1] To approximate time, we calculate the midpoint of the upper and lower bounds of age intervals (e.g., 67 for the age range 65-69). The models included both linear and quadratic time components to capture potential non-linear trajectories. Individual growth trajectories within each class in LCGA were considered homogeneous, and no random effects were fitted.

We explored LCGA with 1 to 9 classes to identify potential subgroups among participants. Maximum likelihood estimation was used for model fitting, with each model rerun 100 times using different initial values to avoid convergence to local maxima. Sample R syntax is provided below:

```
library(lcmm)
set.seed(123)
Model_1class <- lcmm(sleep_category ~ SleepAge + I(SleepAge^2),
            subject = "ID",
            data = dat,
            link = "thresholds",
            maxiter = 100,
            ng = 1)
Model_9class <- gridsearch(rep = 100, maxiter = 100, minit = Model_1class,
                lcmm(sleep_category ~ SleepAge + I(SleepAge^2),
                    mixture = ~ SleepAge + I(SleepAge^2),
                    subject = "ID",
                    data = dat,
                    link = "thresholds",
                    ng = 9))
```

The analysis included participants with complete sleep duration data across the age ranges (18–29 to 75–79 for PPMI-Online and 12–17 to 56–65 for FI). Participants aged "80 or older" and "66 or older" were excluded due to challenges in determining specific ages. Those who were too young to have sleep duration data for all required age periods, or selected "prefer not to answer" or "not sure," were also excluded to minimize violations of the "missing at random" assumption. All participants, regardless of data completeness, were assigned to the class with the highest membership probability.

Model selection was guided by lower Bayesian Information Criteria (BIC), which indicates better model fit, and by interpretability. For both the PPMI-Online and the FI studies, BIC values consistently decreased as the number of classes increased from 1 to 9. We selected the 9-class models for PPMI-Online and the 7-class model for FI, as additional trajectories beyond 7 showed highly similar patterns with no meaningful differences.

For PPMI-Online, trajectory patterns for 1-9 classes are shown in **Figure 1**, and goodness-of-fit statistics are presented in **Figure 2**. Model adequacy metrics, summarized in **Table 1**, were satisfactory, with an average posterior probability of assignments >70%, odds of correct classification >5.0 in all classes, and relative entropy >0.7[2]. The top five most frequent trajectory pattern for each group are presented in **Table 2**. Trajectories from different participant groups are presented in **Figure 3**. For FI, trajectory patterns for 1-9 classes are shown in **Figure 4**, and goodness-of-fit statistics are presented in **Figure 5**. Model adequacy metrics are summarized in **Table 3.** The top five most frequent trajectory pattern for each group are presented in **Table 4**.

We followed the Guidelines for Reporting on Latent Trajectory Studies[3] (checklist on **Table 5**).

References

1. Proust-Lima C, Philipps V, Liquet B. Estimation of Extended Mixed Models Using Latent Classes and Latent Processes: The R Package lcmm. *Journal of Statistical Software*. 2017;78:1-56. doi:10.18637/jss.v078.i02
2. Lennon H, Kelly S, Sperrin M, et al. Framework to construct and interpret latent class trajectory modelling. *BMJ Open*. 2018;8(7):e020683. doi:10.1136/bmjopen-2017-020683
3. Van De Schoot R, Sijbrandij M, Winter SD, Depaoli S, Vermunt JK. The GRoLTS-Checklist: Guidelines for Reporting on Latent Trajectory Studies. *Structural Equation Modeling*. 2017;24(3):451-467. doi:10.1080/10705511.2016.1247646

Supplementary Figure 1. Class-Specific Mean Trajectory Plot of Models (PPMI-Online)

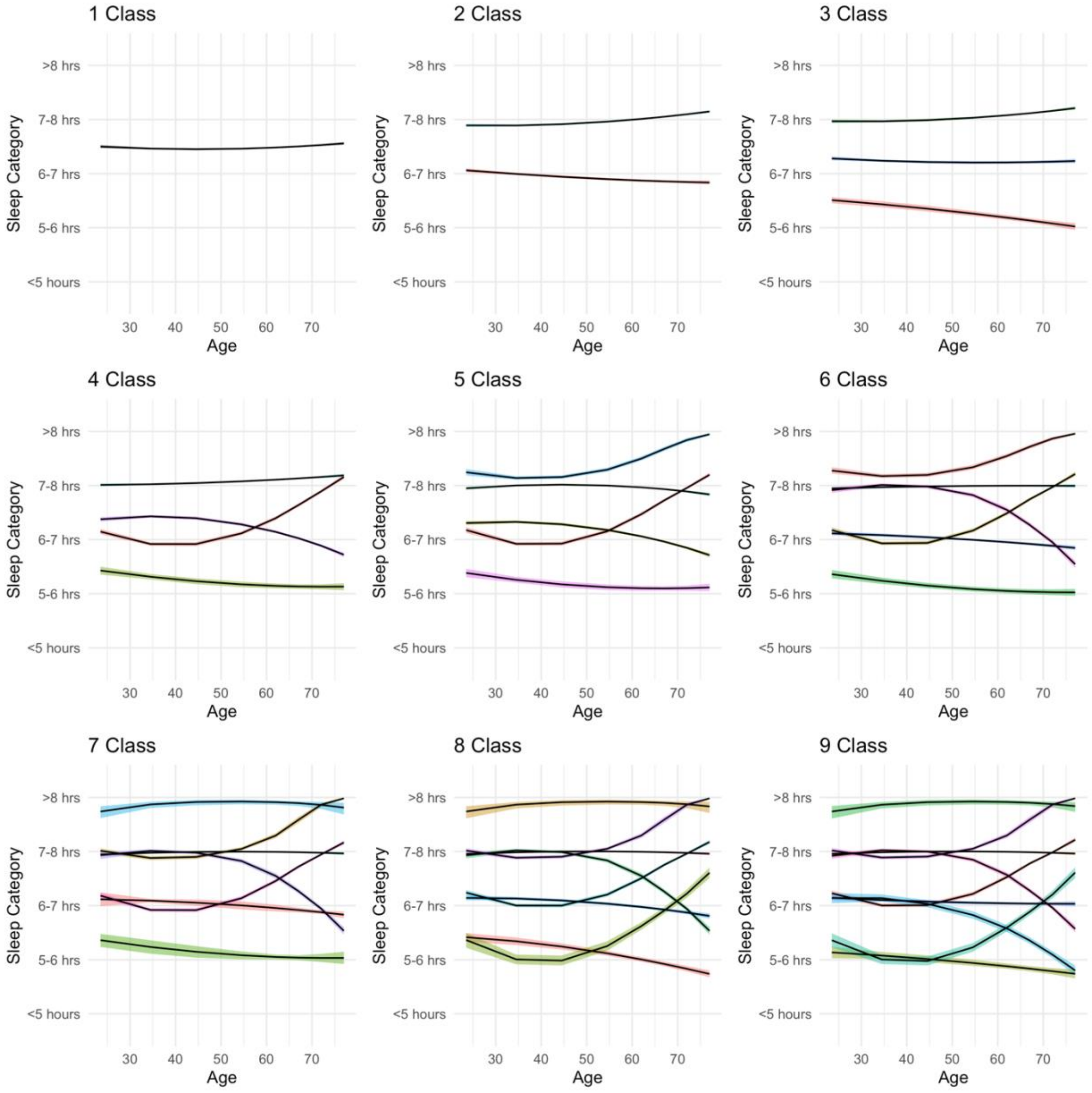

Supplementary Figure 2. Goodness-of-Fit Statistics of Models with 1-9 Classes (PPMI-Online)

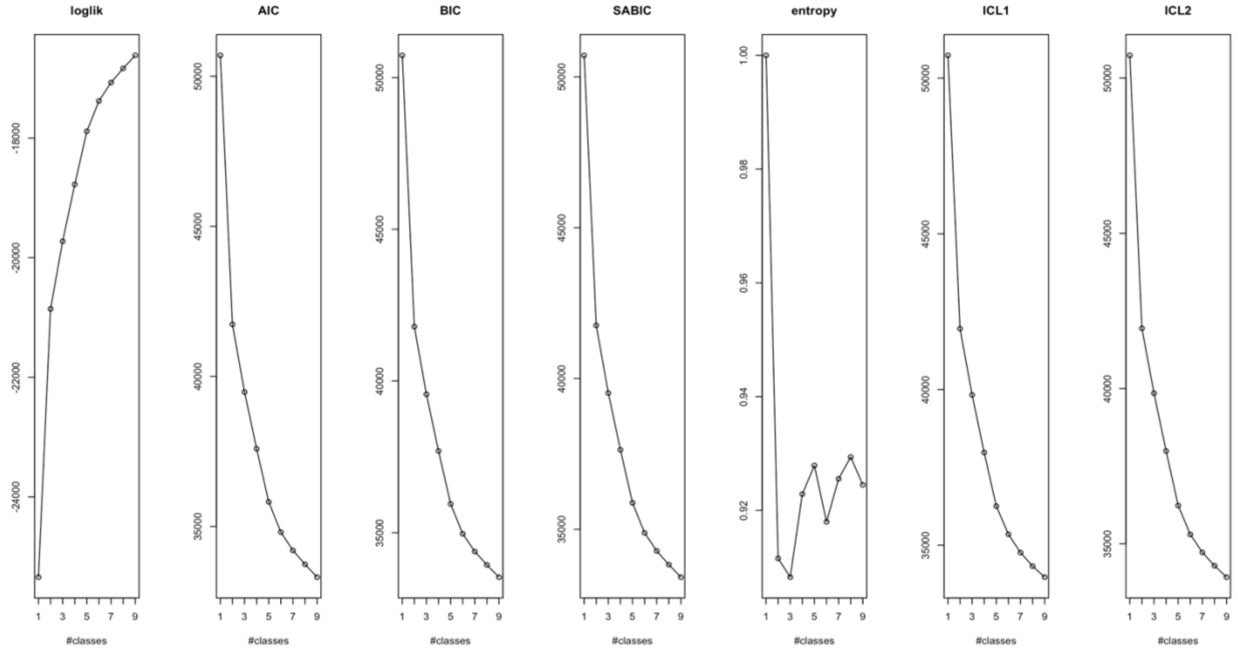

Loglik: log-likelihood, AIC: Akaike Information Criteria, BIC: Bayesian Information Criteria, SABIC: sample-size-adjusted Bayesian Information Criteria

Supplementary Figure 3. Sensitivity Analyses of Sleep Duration Trajectories (PPMI-Online)

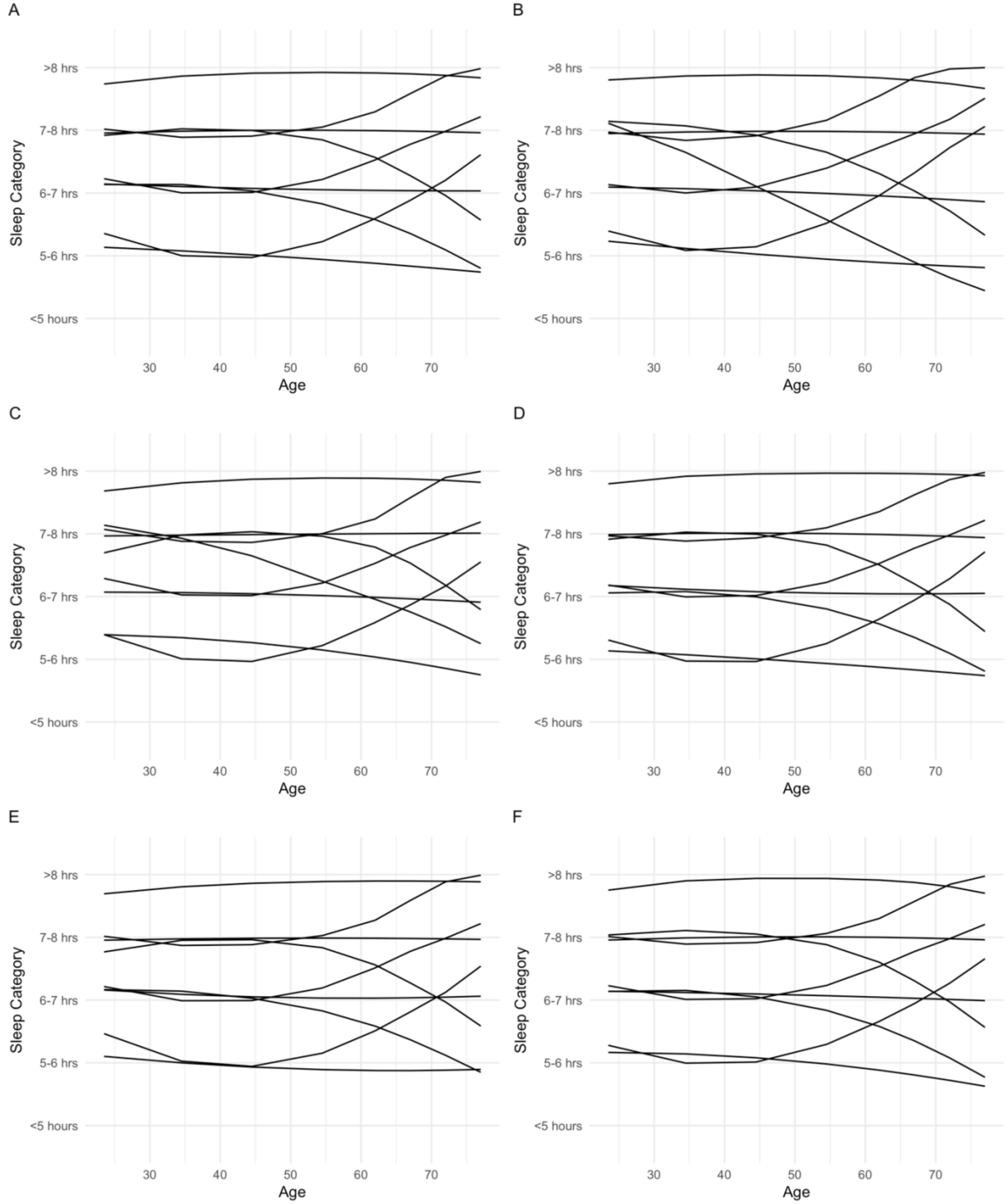

A: Trajectory from participants with complete sleep data (used in our main analysis).
B: Trajectory from all participants.
C: Trajectory from participants not endorsing RBD symptoms and diagnoses throughout follow-up visits.

D: Trajectory from participants endorsing at least one RBD symptoms and diagnoses throughout follow-up visits.
E: Trajectory from participants without PD
F: Trajectory from participants with PD
Comparing the trajectory from participants with complete sleep data (used in our main analysis) (3A) with trajectory from all participants (3B), resulting trajectories were largely similar, except for a group with a decreasing trend starting earlier in life. This likely reflects the reduced number of valid responses after age 50, leading the model to emphasize changes in early adulthood while underestimating changes in later life. The RBD negative (3C) and positive (3D) trajectories look mostly similar, while differences are observed in the patterns previously labeled "≤6 stable" and "6-7 decrease." In negative group, fewer individuals follow the "≤6 stable" and "6-7 decrease" patterns, and a merging of these patterns occurred, forming a new trajectory that have a moderate decline in between 6–7 hrs and 5-6 hrs. This group accounts for 6.3% of individuals. A new trajectory shows a continuous decline in sleep duration, comprising 4.2% of the population. Trajectory from participants without PD (3E) and participants with PD (3F) were largely consistent, suggesting no notable disease-related shapes.

Supplementary Figure 4. Class-Specific Mean Trajectory Plot (FI)

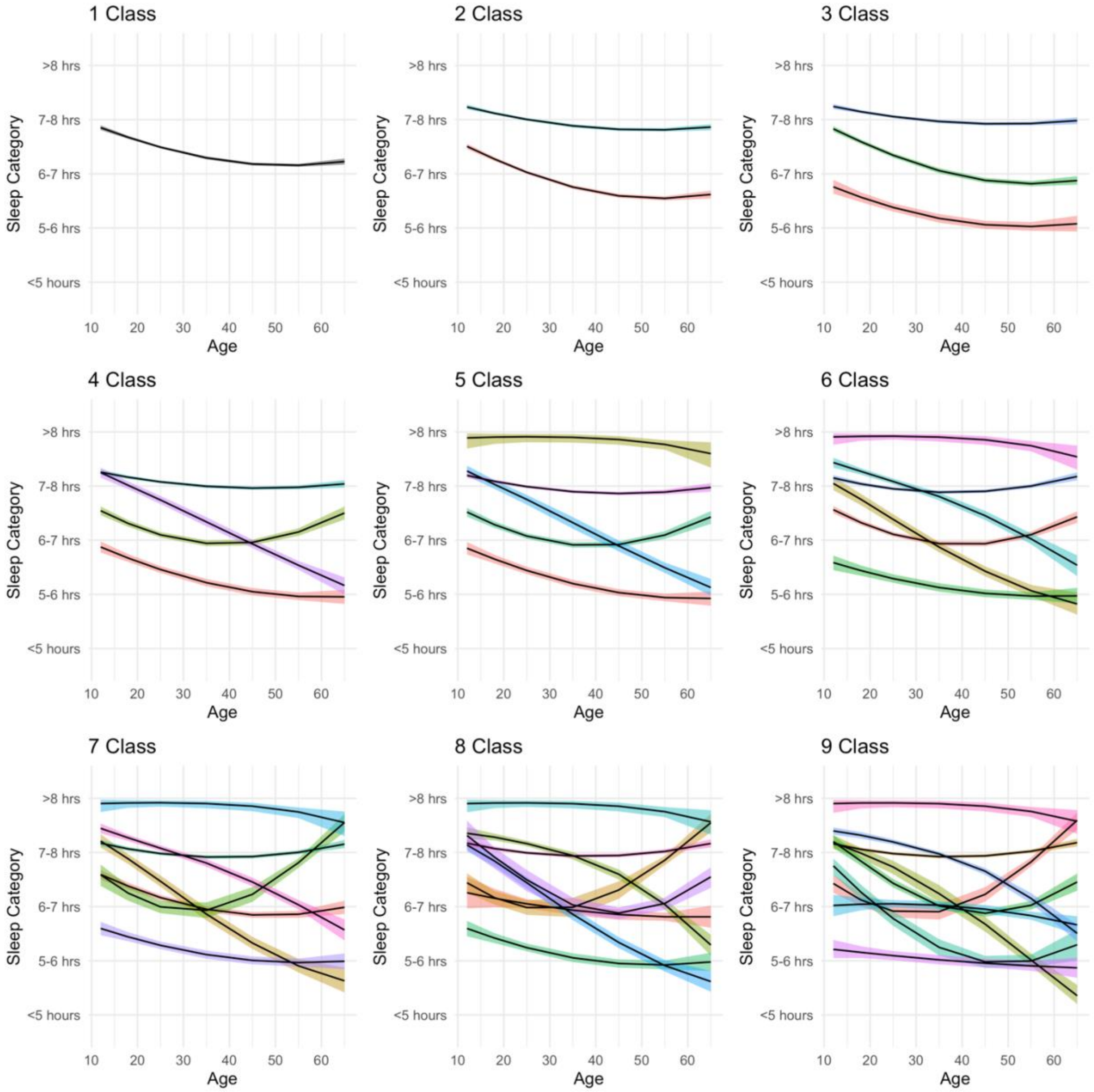

Supplementary Figure 5. Goodness-of-Fit Statistics of Models with 1-9 Classes (FI)

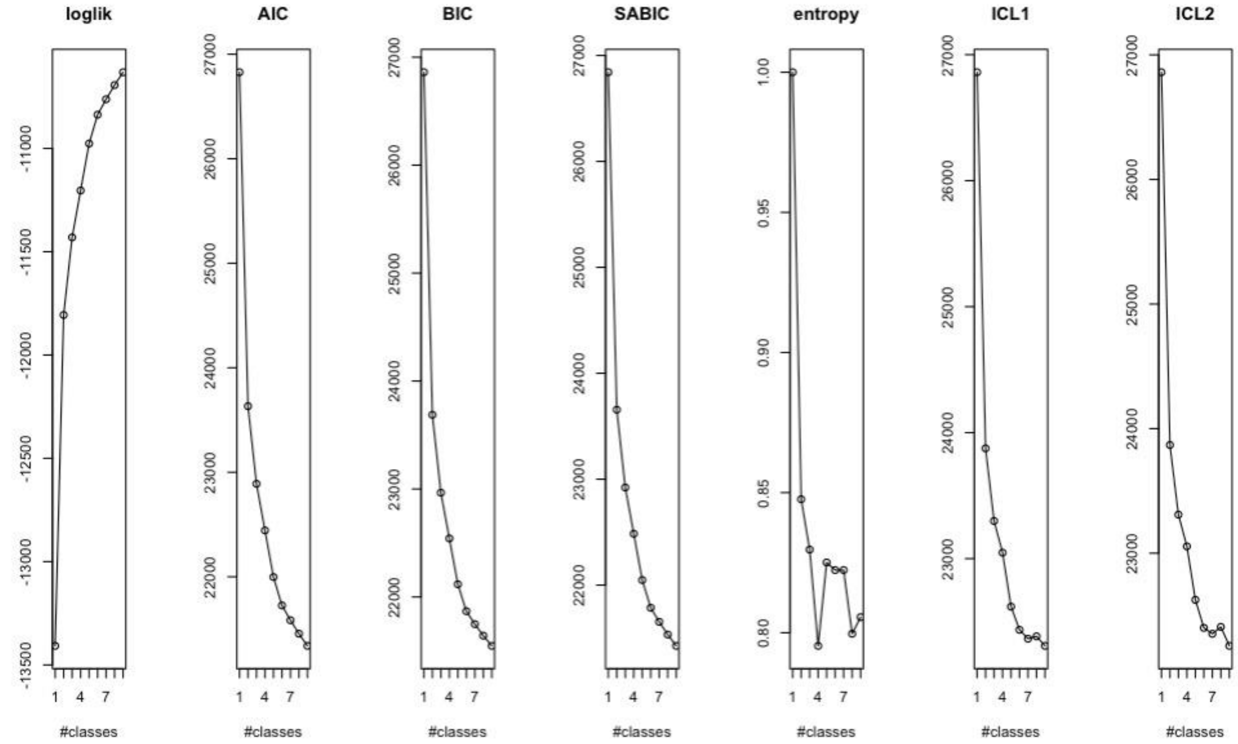

Loglik: log-likelihood, AIC: Akaike Information Criteria, BIC: Bayesian Information Criteria, SABIC: sample-size-adjusted Bayesian Information Criteria

Supplementary Table1. Model Adequacy Measurement of the Model (PPMI-Online)

	<=6 hrs stable	<=6 hrs increase	6-7 hrs decrease	6-7 hrs stable	6-7 hrs increase	7-8 hrs decrease	7-8 hrs stable	7-8 hrs increase	>8 hrs stable
APPA	0.960	0.885	0.891	0.938	0.928	0.910	0.954	0.964	0.954
OCC	429.716	214.326	124.084	60.996	77.435	91.231	47.179	309.484	809.496
Mismatch	0.000	0.002	0.000	-0.005	-0.001	0.003	0.005	-0.004	0.001

APPA: average posterior probability of assignments, OCC: odds of correct classification

Supplementary Table 2. Top 5 Most Frequent Patterns in Each Trajectory Group (PPMI-Online)

Participants with complete data

Trajectory group	Sleep duration pattern	Count	Total	Proportion
	5-6 hrs-5-6 hrs-5-6 hrs-5-6 hrs-5-6 hrs-5-6 hrs-5-6 hrs-5-6 hrs	41		0.295
	6-7 hrs-5-6 hrs-5-6 hrs-5-6 hrs-5-6 hrs-5-6 hrs-5-6 hrs-5-6 hrs	6		0.043
<=6 hrs stable	5-6 hrs-6-7 hrs-5-6 hrs-5-6 hrs-5-6 hrs-5-6 hrs-5-6 hrs-5-6 hrs	5	139	0.036
	<5 hrs-<5 hrs-<5 hrs-<5 hrs-<5 hrs-<5 hrs-<5 hrs-<5 hrs	5		0.036
	5-6 hrs-5-6 hrs-5-6 hrs-5-6 hrs-5-6 hrs-5-6 hrs-5-6 hrs-<5 hrs	4		0.029
	5-6 hrs-5-6 hrs-5-6 hrs-5-6 hrs-6-7 hrs-6-7 hrs-6-7 hrs-6-7 hrs	6		0.063
	5-6 hrs-5-6 hrs-5-6 hrs-6-7 hrs-6-7 hrs-6-7 hrs-7-8 hrs-7-8 hrs	6		0.063
<=6 hrs increase	6-7 hrs-5-6 hrs-5-6 hrs-5-6 hrs-5-6 hrs-6-7 hrs-6-7 hrs-6-7 hrs	6	96	0.063
	6-7 hrs-5-6 hrs-5-6 hrs-6-7 hrs-6-7 hrs-6-7 hrs-6-7 hrs-6-7 hrs	6		0.063
	5-6 hrs-5-6 hrs-5-6 hrs-5-6 hrs-5-6 hrs-5-6 hrs-6-7 hrs-7-8 hrs	4		0.042
	6-7 hrs-6-7 hrs-6-7 hrs-6-7 hrs-6-7 hrs-6-7 hrs-6-7 hrs-6-7 hrs	209		0.411
	7-8 hrs-7-8 hrs-6-7 hrs-6-7 hrs-6-7 hrs-6-7 hrs-6-7 hrs-6-7 hrs	29		0.057
6-7 hrs stable	7-8 hrs-6-7 hrs-6-7 hrs-6-7 hrs-6-7 hrs-6-7 hrs-6-7 hrs-6-7 hrs	28	508	0.055
	6-7 hrs-6-7 hrs-6-7 hrs-6-7 hrs-6-7 hrs-6-7 hrs-6-7 hrs-7-8 hrs	24		0.047
	6-7 hrs-6-7 hrs-6-7 hrs-6-7 hrs-6-7 hrs-6-7 hrs-6-7 hrs-5-6 hrs	21		0.041
	6-7 hrs-6-7 hrs-6-7 hrs-6-7 hrs-6-7 hrs-6-7 hrs-5-6 hrs-5-6 hrs	24		0.146
	6-7 hrs-6-7 hrs-6-7 hrs-5-6 hrs-5-6 hrs-5-6 hrs-5-6 hrs-5-6 hrs	14		0.085
	6-7 hrs-6-7 hrs-6-7 hrs-6-7 hrs-6-7 hrs-5-6 hrs-5-6 hrs-5-6 hrs	10		0.061
6-7 hrs decrease	6-7 hrs-6-7 hrs-6-7 hrs-6-7 hrs-5-6 hrs-5-6 hrs-5-6 hrs-5-6 hrs	9	164	0.055
	5-6 hrs-6-7 hrs-6-7 hrs-6-7 hrs-6-7 hrs-5-6 hrs-5-6 hrs-5-6 hrs	5		0.030
	5-6 hrs-6-7 hrs-6-7 hrs-6-7 hrs-6-7 hrs-6-7 hrs-5-6 hrs-5-6 hrs	5		0.030
	7-8 hrs-7-8 hrs-6-7 hrs-6-7 hrs-6-7 hrs-6-7 hrs-5-6 hrs-5-6 hrs	5		0.030
	6-7 hrs-6-7 hrs-6-7 hrs-6-7 hrs-6-7 hrs-7-8 hrs-7-8 hrs-7-8 hrs	33		0.088
	6-7 hrs-6-7 hrs-6-7 hrs-6-7 hrs-6-7 hrs-6-7 hrs-7-8 hrs-7-8 hrs	32		0.086
6-7 hrs increase	6-7 hrs-6-7 hrs-6-7 hrs-7-8 hrs-7-8 hrs-7-8 hrs-7-8 hrs-7-8 hrs	32	373	0.086
	6-7 hrs-6-7 hrs-6-7 hrs-6-7 hrs-7-8 hrs-7-8 hrs-7-8 hrs-7-8 hrs	28		0.075
	7-8 hrs-6-7 hrs-6-7 hrs-6-7 hrs-7-8 hrs-7-8 hrs-7-8 hrs-7-8 hrs	19		0.051
	7-8 hrs-7-8 hrs-7-8 hrs-7-8 hrs-7-8 hrs-7-8 hrs-7-8 hrs-7-8 hrs	526		0.641
	7-8 hrs-7-8 hrs-7-8 hrs-7-8 hrs-7-8 hrs-7-8 hrs-7-8 hrs-6-7 hrs	41		0.050
7-8 hrs stable	6-7 hrs-7-8 hrs-7-8 hrs-7-8 hrs-7-8 hrs-7-8 hrs-7-8 hrs-7-8 hrs	35	821	0.043
	7-8 hrs-7-8 hrs-7-8 hrs-7-8 hrs-7-8 hrs-7-8 hrs-7-8 hrs->8 hrs	32		0.039
	6-7 hrs-6-7 hrs-7-8 hrs-7-8 hrs-7-8 hrs-7-8 hrs-7-8 hrs-7-8 hrs	22		0.027
	7-8 hrs-7-8 hrs-7-8 hrs-7-8 hrs-7-8 hrs-7-8 hrs-6-7 hrs-6-7 hrs	43		0.158
7-8 hrs decrease	7-8 hrs-7-8 hrs-7-8 hrs-7-8 hrs-6-7 hrs-6-7 hrs-6-7 hrs-6-7 hrs	34	273	0.125
	7-8 hrs-7-8 hrs-7-8 hrs-6-7 hrs-6-7 hrs-6-7 hrs-6-7 hrs-6-7 hrs	17		0.062
	7-8 hrs-7-8 hrs-7-8 hrs-7-8 hrs-7-8 hrs-6-7 hrs-6-7 hrs-6-7 hrs	16		0.059

	7-8 hrs-7-8 hrs-7-8 hrs-7-8 hrs-6-7 hrs-6-7 hrs-6-7 hrs-5-6 hrs	9		0.033
	7-8 hrs-7-8 hrs-7-8 hrs-7-8 hrs-7-8 hrs-7-8 hrs-6-7 hrs-5-6 hrs	9		0.033
	7-8 hrs-7-8 hrs-7-8 hrs-7-8 hrs-7-8 hrs-7-8 hrs-7-8 hrs-5-6 hrs	9		0.033
7-8 hrs increase	7-8 hrs-7-8 hrs-7-8 hrs-7-8 hrs-7-8 hrs-7-8 hrs->8 hrs->8 hrs	47	201	0.234
	7-8 hrs-7-8 hrs-7-8 hrs-7-8 hrs->8 hrs->8 hrs->8 hrs->8 hrs	32		0.159
	7-8 hrs-7-8 hrs-7-8 hrs-7-8 hrs-7-8 hrs->8 hrs->8 hrs->8 hrs	29		0.144
	>8 hrs-7-8 hrs-7-8 hrs-7-8 hrs-7-8 hrs-7-8 hrs->8 hrs->8 hrs	12		0.060
	7-8 hrs-7-8 hrs-7-8 hrs->8 hrs->8 hrs->8 hrs->8 hrs->8 hrs	9		0.045
>8 hrs stable	>8 hrs->8 hrs->8 hrs->8 hrs->8 hrs->8 hrs->8 hrs->8 hrs	37	68	0.544
	7-8 hrs-7-8 hrs->8 hrs->8 hrs->8 hrs->8 hrs->8 hrs->8 hrs	5		0.074
	7-8 hrs->8 hrs->8 hrs->8 hrs->8 hrs->8 hrs->8 hrs->8 hrs	4		0.059
	>8 hrs-7-8 hrs-7-8 hrs->8 hrs->8 hrs->8 hrs->8 hrs->8 hrs	3		0.044
	>8 hrs->8 hrs->8 hrs->8 hrs-7-8 hrs-7-8 hrs-7-8 hrs-7-8 hrs	3		0.044

Participants with incomplete data

Trajectory group	Sleep duration pattern	Count	Total	Proportion
<=6 hrs stable	5-6 hrs-5-6 hrs-5-6 hrs-5-6 hrs-NA-NA-NA-NA	80	1044	0.077
	5-6 hrs-5-6 hrs-5-6 hrs-5-6 hrs-5-6 hrs-5-6 hrs-NA-NA	76		0.073
	5-6 hrs-5-6 hrs-5-6 hrs-5-6 hrs-5-6 hrs-NA-NA-NA	75		0.072
	5-6 hrs-5-6 hrs-5-6 hrs-5-6 hrs-5-6 hrs-5-6 hrs-5-6 hrs-NA	56		0.054
	6-7 hrs-6-7 hrs-5-6 hrs-5-6 hrs-NA-NA-NA-NA	39		0.037
<=6 hrs increase	5-6 hrs-5-6 hrs-6-7 hrs-6-7 hrs-NA-NA-NA-NA	33	622	0.053
	6-7 hrs-5-6 hrs-5-6 hrs-5-6 hrs-NA-NA-NA-NA	33		0.053
	6-7 hrs-5-6 hrs-5-6 hrs-6-7 hrs-NA-NA-NA-NA	31		0.050
	5-6 hrs-5-6 hrs-5-6 hrs-6-7 hrs-NA-NA-NA-NA	24		0.039
	5-6 hrs-5-6 hrs-5-6 hrs-5-6 hrs-6-7 hrs-NA-NA-NA	23		0.037
6-7 hrs stable	6-7 hrs-6-7 hrs-6-7 hrs-6-7 hrs-NA-NA-NA-NA	299	2857	0.105
	6-7 hrs-6-7 hrs-6-7 hrs-6-7 hrs-6-7 hrs-6-7 hrs-NA-NA	286		0.100
	6-7 hrs-6-7 hrs-6-7 hrs-6-7 hrs-6-7 hrs-6-7 hrs-6-7 hrs-NA	254		0.089
	6-7 hrs-6-7 hrs-6-7 hrs-6-7 hrs-6-7 hrs-NA-NA-NA	234		0.082
	7-8 hrs-6-7 hrs-6-7 hrs-6-7 hrs-NA-NA-NA-NA	106		0.037
6-7 hrs decrease	6-7 hrs-6-7 hrs-6-7 hrs-6-7 hrs-5-6 hrs-NA-NA-NA	42	998	0.042
	6-7 hrs-6-7 hrs-6-7 hrs-5-6 hrs-5-6 hrs-NA-NA-NA	34		0.034
	6-7 hrs-6-7 hrs-6-7 hrs-6-7 hrs-5-6 hrs-5-6 hrs-NA-NA	34		0.034
	6-7 hrs-6-7 hrs-6-7 hrs-5-6 hrs-5-6 hrs-5-6 hrs-NA-NA	31		0.031
	7-8 hrs-7-8 hrs-7-8 hrs-6-7 hrs-5-6 hrs-NA-NA-NA	31		0.031
6-7 hrs increase	6-7 hrs-6-7 hrs-6-7 hrs-6-7 hrs-7-8 hrs-7-8 hrs-NA-NA	107	1950	0.055
	6-7 hrs-6-7 hrs-6-7 hrs-7-8 hrs-NA-NA-NA-NA	107		0.055
	6-7 hrs-6-7 hrs-6-7 hrs-6-7 hrs-7-8 hrs-NA-NA-NA	87		0.045
	6-7 hrs-6-7 hrs-6-7 hrs-6-7 hrs-6-7 hrs-7-8 hrs-NA-NA	71		0.036
	7-8 hrs-6-7 hrs-6-7 hrs-7-8 hrs-NA-NA-NA-NA	62		0.032

7-8 hrs stable	7-8 hrs-7-8 hrs-7-8 hrs-7-8 hrs-7-8 hrs-7-8 hrs-NA-NA	587	3405	0.172
	7-8 hrs-7-8 hrs-7-8 hrs-7-8 hrs-7-8 hrs-7-8 hrs-7-8 hrs-NA	512		0.150
	7-8 hrs-7-8 hrs-7-8 hrs-7-8 hrs-NA-NA-NA-NA	481		0.141
	7-8 hrs-7-8 hrs-7-8 hrs-7-8 hrs-7-8 hrs-NA-NA-NA	399		0.117
	7-8 hrs-7-8 hrs-7-8 hrs-6-7 hrs-NA-NA-NA-NA	135		0.040
7-8 hrs decrease	7-8 hrs-7-8 hrs-7-8 hrs-7-8 hrs-6-7 hrs-NA-NA-NA	88	1107	0.079
	7-8 hrs-7-8 hrs-7-8 hrs-7-8 hrs-6-7 hrs-6-7 hrs-NA-NA	77		0.070
	7-8 hrs-7-8 hrs-7-8 hrs-6-7 hrs-6-7 hrs-NA-NA-NA	61		0.055
	7-8 hrs-7-8 hrs-7-8 hrs-7-8 hrs-7-8 hrs-6-7 hrs-NA-NA	59		0.053
	7-8 hrs-7-8 hrs-7-8 hrs-6-7 hrs-6-7 hrs-6-7 hrs-NA-NA	58		0.052
7-8 hrs increase	7-8 hrs-7-8 hrs-7-8 hrs-7-8 hrs-7-8 hrs-7-8 hrs->8 hrs-NA	68	838	0.081
	7-8 hrs-7-8 hrs-7-8 hrs-7-8 hrs->8 hrs->8 hrs-NA-NA	59		0.070
	7-8 hrs-7-8 hrs-7-8 hrs-7-8 hrs->8 hrs-NA-NA-NA	57		0.068
	7-8 hrs-7-8 hrs-7-8 hrs-7-8 hrs-7-8 hrs->8 hrs-NA-NA	56		0.067
	7-8 hrs-7-8 hrs-7-8 hrs-7-8 hrs-7-8 hrs->8 hrs->8 hrs-NA	46		0.055
>8 hrs stable	>8 hrs->8 hrs->8 hrs->8 hrs-NA-NA-NA-NA	63	441	0.143
	>8 hrs->8 hrs->8 hrs->8 hrs->8 hrs->8 hrs->8 hrs-NA	48		0.109
	>8 hrs->8 hrs->8 hrs->8 hrs->8 hrs-NA-NA-NA	45		0.102
	>8 hrs->8 hrs->8 hrs->8 hrs->8 hrs->8 hrs-NA-NA	44		0.100
	7-8 hrs-7-8 hrs->8 hrs->8 hrs-NA-NA-NA-NA	26		0.059

The patterns are ordered as follows: 18–29, 30–39, 40–49, 50–59, 60–64, 65–69, 70–74, and 75–79.

Supplementary Table 3. Model Adequacy Measurement of the Model (FI)

	<= 6 hrs	6-7 hrs	7-8 hrs	> 8 hrs	decrease-1	decrease-2	increase
%	10.96	28.88	28.82	2.58	7.92	15.06	5.79
APPA	0.909	0.844	0.918	0.966	0.792	0.796	0.812
OCC	80.523	13.804	27.617	1046.252	43.167	23.750	57.530
Mismatch	-0.001	0.007	-0.001	0.000	-0.002	0.010	-0.012

APPA: average posterior probability of assignments, OCC: odds of correct classification

Supplementary Table 4. Top 5 Most Frequent Patterns in Each Trajectory Group (FI)

Participants with complete data

Trajectory group	Sleep duration pattern	Count	Total	Proportion
	5-6 hrs-5-6 hrs-5-6 hrs-5-6 hrs-5-6 hrs-5-6 hrs	26		0.149
	6-7 hrs-5-6 hrs-5-6 hrs-5-6 hrs-5-6 hrs-5-6 hrs	16		0.092
<=6 hrs	6-7 hrs-6-7 hrs-6-7 hrs-5-6 hrs-5-6 hrs-5-6 hrs	12	174	0.069
	6-7 hrs-6-7 hrs-5-6 hrs-5-6 hrs-5-6 hrs-5-6 hrs	8		0.046
	7-8 hrs-5-6 hrs-5-6 hrs-5-6 hrs-5-6 hrs-5-6 hrs	6		0.034
	7-8 hrs-6-7 hrs-6-7 hrs-6-7 hrs-6-7 hrs-6-7 hrs	77		0.157
	6-7 hrs-6-7 hrs-6-7 hrs-6-7 hrs-6-7 hrs-6-7 hrs	63		0.129
6-7 hrs	7-8 hrs-7-8 hrs-6-7 hrs-6-7 hrs-6-7 hrs-6-7 hrs	48	489	0.098
	7-8 hrs-6-7 hrs-6-7 hrs-6-7 hrs-6-7 hrs-5-6 hrs	29		0.059
	6-7 hrs-6-7 hrs-6-7 hrs-6-7 hrs-6-7 hrs-5-6 hrs	17		0.035
	7-8 hrs-7-8 hrs-6-7 hrs-6-7 hrs-6-7 hrs-5-6 hrs	17		0.035
	7-8 hrs-7-8 hrs-7-8 hrs-7-8 hrs-7-8 hrs-7-8 hrs	207		0.416
	>8 hrs-7-8 hrs-7-8 hrs-7-8 hrs-7-8 hrs-7-8 hrs	58		0.116
7-8 hrs	7-8 hrs-7-8 hrs-7-8 hrs-7-8 hrs-7-8 hrs->8 hrs	23	498	0.046
	7-8 hrs-6-7 hrs-7-8 hrs-7-8 hrs-7-8 hrs-7-8 hrs	17		0.034
	7-8 hrs-7-8 hrs-7-8 hrs-6-7 hrs-6-7 hrs-7-8 hrs	12		0.024
	>8 hrs->8 hrs->8 hrs->8 hrs->8 hrs->8 hrs	18		0.429
	>8 hrs->8 hrs->8 hrs->8 hrs->8 hrs-7-8 hrs	6		0.143
>8 hrs	>8 hrs->8 hrs->8 hrs->8 hrs-7-8 hrs-7-8 hrs	4	42	0.095
	>8 hrs-7-8 hrs->8 hrs->8 hrs->8 hrs->8 hrs	2		0.048
	>8 hrs->8 hrs-7-8 hrs-7-8 hrs->8 hrs->8 hrs	2		0.048
	>8 hrs->8 hrs->8 hrs-7-8 hrs->8 hrs->8 hrs	2		0.048
	7-8 hrs-6-7 hrs-6-7 hrs-6-7 hrs-7-8 hrs-7-8 hrs	13		0.133
	6-7 hrs-6-7 hrs-6-7 hrs-6-7 hrs-7-8 hrs-7-8 hrs	9		0.092
increase	6-7 hrs-6-7 hrs-6-7 hrs-6-7 hrs-6-7 hrs-7-8 hrs	8	98	0.082
	6-7 hrs-6-7 hrs-6-7 hrs-7-8 hrs-7-8 hrs-7-8 hrs	6		0.061
	6-7 hrs-5-6 hrs-6-7 hrs-6-7 hrs-6-7 hrs-7-8 hrs	4		0.041
	7-8 hrs-6-7 hrs-6-7 hrs-6-7 hrs-5-6 hrs-5-6 hrs	15		0.128
	7-8 hrs-6-7 hrs-5-6 hrs-5-6 hrs-5-6 hrs-5-6 hrs	10		0.085
decrease-1	7-8 hrs-7-8 hrs-6-7 hrs-6-7 hrs-5-6 hrs-<5 hrs	7	117	0.060
	7-8 hrs-6-7 hrs-6-7 hrs-5-6 hrs-5-6 hrs-5-6 hrs	5		0.043
	7-8 hrs-7-8 hrs-6-7 hrs-6-7 hrs-5-6 hrs-5-6 hrs	5		0.043
	7-8 hrs-7-8 hrs-7-8 hrs-7-8 hrs-7-8 hrs-6-7 hrs	35		0.140
	7-8 hrs-7-8 hrs-7-8 hrs-7-8 hrs-6-7 hrs-6-7 hrs	26		0.104
decrease-2	7-8 hrs-7-8 hrs-7-8 hrs-6-7 hrs-6-7 hrs-6-7 hrs	17	250	0.068
	>8 hrs-7-8 hrs-7-8 hrs-7-8 hrs-7-8 hrs-6-7 hrs	15		0.060
	7-8 hrs-7-8 hrs-7-8 hrs-7-8 hrs-7-8 hrs-5-6 hrs	12		0.048

Participants with incomplete data

Trajectory group	Sleep duration pattern	Count	Total	Proportion
	5-6 hrs-5-6 hrs-5-6 hrs-NA-NA-NA	2		0.059
	5-6 hrs-NA-NA-NA-NA-NA	2		0.059
	NA-5-6 hrs-5-6 hrs-5-6 hrs-5-6 hrs-5-6 hrs	2		0.059
	5-6 hrs-5-6 hrs-5-6 hrs-5-6 hrs-5-6 hrs-NA	1		0.029
	5-6 hrs-5-6 hrs-5-6 hrs-5-6 hrs-<5 hrs-NA	1		0.029
	5-6 hrs-5-6 hrs-<5 hrs-<5 hrs-<5 hrs-NA	1		0.029
	5-6 hrs-6-7 hrs-6-7 hrs-6-7 hrs-5-6 hrs-NA	1		0.029
	5-6 hrs-<5 hrs-<5 hrs-<5 hrs-7-8 hrs-NA	1		0.029
	5-6 hrs-NA-NA-6-7 hrs-6-7 hrs-5-6 hrs	1		0.029
	6-7 hrs-5-6 hrs-5-6 hrs-5-6 hrs-5-6 hrs-NA	1		0.029
	6-7 hrs-5-6 hrs-6-7 hrs-6-7 hrs-5-6 hrs-NA	1		0.029
	6-7 hrs-5-6 hrs-<5 hrs-<5 hrs-<5 hrs-NA	1		0.029
	6-7 hrs-6-7 hrs-5-6 hrs-5-6 hrs-<5 hrs-NA	1		0.029
	6-7 hrs-6-7 hrs-6-7 hrs-5-6 hrs-<5 hrs-NA	1		0.029
	6-7 hrs-NA-NA-NA-NA-5-6 hrs	1		0.029
<=6 hrs	7-8 hrs-5-6 hrs-5-6 hrs-5-6 hrs-5-6 hrs-NA	1	34	0.029
	7-8 hrs-5-6 hrs-NA-5-6 hrs-<5 hrs-5-6 hrs	1		0.029
	<5 hrs-5-6 hrs-NA-NA-NA-NA	1		0.029
	<5 hrs-NA-NA-NA-NA-NA	1		0.029
	>8 hrs-5-6 hrs-<5 hrs-<5 hrs-<5 hrs-NA	1		0.029
	NA-5-6 hrs-5-6 hrs-5-6 hrs-5-6 hrs-6-7 hrs	1		0.029
	NA-5-6 hrs-<5 hrs-5-6 hrs-5-6 hrs-6-7 hrs	1		0.029
	NA-5-6 hrs-NA-NA-5-6 hrs-5-6 hrs	1		0.029
	NA-6-7 hrs-5-6 hrs-5-6 hrs-5-6 hrs-5-6 hrs	1		0.029
	NA-6-7 hrs-5-6 hrs-5-6 hrs-5-6 hrs-6-7 hrs	1		0.029
	NA-6-7 hrs-5-6 hrs-5-6 hrs-<5 hrs-<5 hrs	1		0.029
	NA-6-7 hrs-<5 hrs-5-6 hrs-5-6 hrs-5-6 hrs	1		0.029
	NA-NA-5-6 hrs-5-6 hrs-5-6 hrs-6-7 hrs	1		0.029
	NA-NA-NA-5-6 hrs-5-6 hrs-5-6 hrs	1		0.029
	NA-NA-NA-5-6 hrs-<5 hrs-6-7 hrs	1		0.029
	NA-NA-NA-5-6 hrs-<5 hrs-<5 hrs	1		0.029
	NA-NA-NA-NA-NA-6-7 hrs	8		0.105
	7-8 hrs-6-7 hrs-6-7 hrs-6-7 hrs-5-6 hrs-NA	7		0.092
6-7 hrs	7-8 hrs-6-7 hrs-6-7 hrs-6-7 hrs-6-7 hrs-NA	5	76	0.066
	6-7 hrs-6-7 hrs-6-7 hrs-6-7 hrs-5-6 hrs-NA	4		0.053
	6-7 hrs-6-7 hrs-6-7 hrs-6-7 hrs-6-7 hrs-NA	4		0.053
	6-7 hrs-NA-NA-NA-NA-NA	4		0.053
7-8 hrs	7-8 hrs-NA-NA-NA-NA-NA	8	65	0.123

	7-8 hrs-7-8 hrs-7-8 hrs-7-8 hrs-7-8 hrs-NA	6		0.092
	7-8 hrs-7-8 hrs-NA-NA-NA-NA	6		0.092
	NA-7-8 hrs-7-8 hrs-7-8 hrs-7-8 hrs-7-8 hrs	5		0.077
	NA-NA-7-8 hrs-7-8 hrs-7-8 hrs-7-8 hrs	4		0.062
	NA-NA-NA-NA-7-8 hrs-7-8 hrs	4		0.062
>8 hrs	>8 hrs->8 hrs-NA-NA-NA-NA	4	11	0.364
	>8 hrs->8 hrs->8 hrs-7-8 hrs-7-8 hrs-NA	2		0.182
	NA->8 hrs->8 hrs->8 hrs->8 hrs->8 hrs	2		0.182
	7-8 hrs->8 hrs->8 hrs->8 hrs->8 hrs-NA	1		0.091
	>8 hrs-NA-NA-NA-NA->8 hrs	1		0.091
	NA-NA->8 hrs-NA-NA-NA	1		0.091
increase	6-7 hrs-6-7 hrs-6-7 hrs-6-7 hrs-7-8 hrs-NA	3	14	0.214
	NA-6-7 hrs-6-7 hrs-6-7 hrs-6-7 hrs-7-8 hrs	2		0.143
	6-7 hrs-6-7 hrs-6-7 hrs-6-7 hrs-NA-7-8 hrs	1		0.071
	6-7 hrs-6-7 hrs-6-7 hrs-7-8 hrs-7-8 hrs-NA	1		0.071
	6-7 hrs-6-7 hrs-6-7 hrs-NA-7-8 hrs-7-8 hrs	1		0.071
	6-7 hrs-NA-NA-NA-7-8 hrs-7-8 hrs	1		0.071
	7-8 hrs-5-6 hrs-5-6 hrs-5-6 hrs-7-8 hrs-NA	1		0.071
	<5 hrs-<5 hrs-7-8 hrs-NA-7-8 hrs->8 hrs	1		0.071
	NA-5-6 hrs-6-7 hrs-6-7 hrs-7-8 hrs-7-8 hrs	1		0.071
	NA-5-6 hrs-6-7 hrs-7-8 hrs-7-8 hrs-7-8 hrs	1		0.071
	NA-6-7 hrs-5-6 hrs-6-7 hrs-6-7 hrs-7-8 hrs	1		0.071
decrease-1	7-8 hrs-6-7 hrs-6-7 hrs-5-6 hrs-5-6 hrs-NA	4	22	0.182
	7-8 hrs-7-8 hrs-6-7 hrs-6-7 hrs-5-6 hrs-NA	4		0.182
	7-8 hrs-7-8 hrs-7-8 hrs-6-7 hrs-5-6 hrs-NA	2		0.091
	>8 hrs-6-7 hrs-6-7 hrs-5-6 hrs-6-7 hrs-NA	2		0.091
	7-8 hrs-6-7 hrs-6-7 hrs-6-7 hrs-<5 hrs-NA	1		0.045
	7-8 hrs-7-8 hrs-5-6 hrs-<5 hrs-<5 hrs-NA	1		0.045
	7-8 hrs-7-8 hrs-6-7 hrs-5-6 hrs-5-6 hrs-NA	1		0.045
	7-8 hrs-7-8 hrs-6-7 hrs-6-7 hrs-<5 hrs-NA	1		0.045
	7-8 hrs-7-8 hrs-7-8 hrs-5-6 hrs-NA-5-6 hrs	1		0.045
	7-8 hrs-NA-6-7 hrs-<5 hrs-<5 hrs-5-6 hrs	1		0.045
	>8 hrs-NA-6-7 hrs-6-7 hrs-<5 hrs-NA	1		0.045
	NA-7-8 hrs-7-8 hrs-6-7 hrs-6-7 hrs-<5 hrs	1		0.045
	NA-NA-7-8 hrs-6-7 hrs-5-6 hrs-<5 hrs	1		0.045
	NA-NA-NA-NA-5-6 hrs-<5 hrs	1		0.045
decrease-2	7-8 hrs-7-8 hrs-7-8 hrs-7-8 hrs-6-7 hrs-NA	5	39	0.128
	>8 hrs-NA-NA-NA-NA-NA	4		0.103
	NA-7-8 hrs-7-8 hrs-7-8 hrs-7-8 hrs-6-7 hrs	4		0.103
	>8 hrs-7-8 hrs-6-7 hrs-6-7 hrs-6-7 hrs-NA	2		0.051

>8 hrs->8 hrs-7-8 hrs-7-8 hrs-5-6 hrs-NA	2	0.051
>8 hrs->8 hrs-7-8 hrs-7-8 hrs-6-7 hrs-NA	2	0.051
>8 hrs->8 hrs->8 hrs-7-8 hrs-6-7 hrs-NA	2	0.051
NA-NA-NA-7-8 hrs-7-8 hrs-6-7 hrs	2	0.051

The patterns are ordered as follows: 12-17, 18-25, 26-35, 36-45, 46-55, and 56-65

Supplementary Table 5. Guidelines for Reporting on Latent Trajectory Studies (GRoLTS) Checklist

	Checklist Item	Reported?
1	Is the metric of time used in the statistical model reported?	Yes
2	Is information presented about the mean and variance of time within a wave?	Not Applicable
3a	Is the missing data mechanism reported?	Yes
3b	Is a description provided of what variables are related to attrition/missing data?	Yes
3c	Is a description provided of how missing data in the analyses were dealt with?	Yes
4	Is information about the distribution of the observed variables included?	Yes
5	Is the software mentioned?	Yes
6a	Are alternative specifications of within-class heterogeneity considered (e.g., LGCA vs. LGMM) and clearly documented? If not, was sufficient justification provided as to eliminate certain specifications from consideration?	Yes
6b	Are alternative specifications of the between-class differences in variance–covariance matrix structure considered and clearly documented? If not, was sufficient justification provided as to eliminate certain specifications from consideration?	Yes
7	Are alternative shape/functional forms of the trajectories described?	Yes
8	If covariates have been used, can analyses still be replicated?	No
9	Is information reported about the number of random start values and final iterations included?	Yes
10	Are the model comparison (and selection) tools described from a statistical perspective?	Yes
11	Are the total number of fitted models reported, including a one-class solution?	Yes
12	Are the number of cases per class reported for each model (absolute sample size, or proportion)?	Yes
13	If classification of cases in a trajectory is the goal, is entropy reported?	Yes
14a	Is a plot included with the estimated mean trajectories of the final solution?	Yes
14b	Are plots included with the estimated mean trajectories for each model?	Yes
14c	Is a plot included of the combination of estimated means of the final model and the observed individual trajectories split out for each latent class?	Yes
15	Are characteristics of the final class solution numerically described (i.e., means, SD/SE, n, CI, etc.)?	Yes
16	Are the syntax files available (either in the appendix, supplementary materials, or from the authors)?	Yes

Supplementary Figure 1. PPMI-Online Study Sensitivity Analysis - 1
Participants endorsed history of RBD symptoms or diagnoses during any of the follow-up excluded

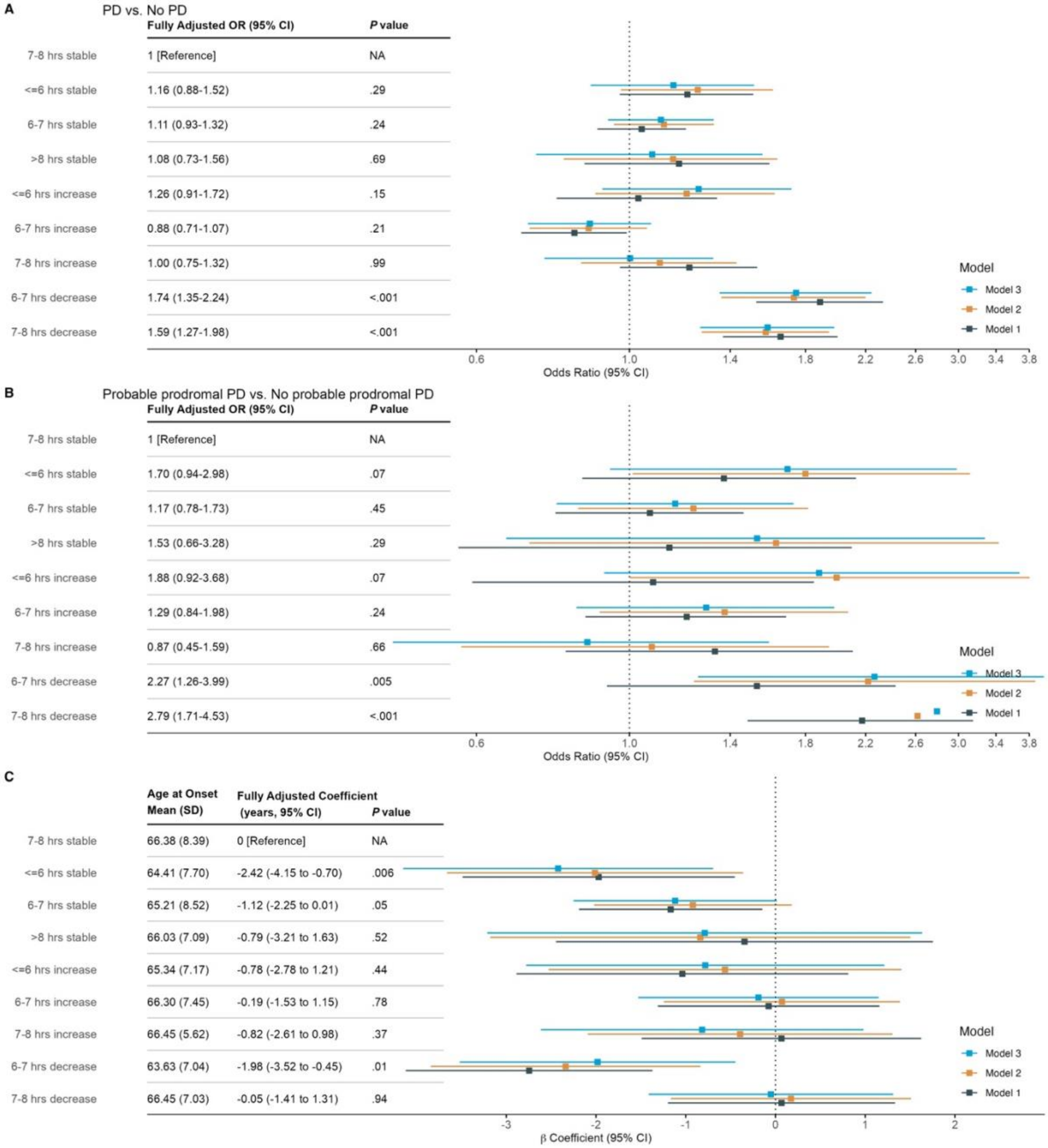

Abbreviations: OR = Odds ratio, pPD = prodromal Parkinson's disease.
Panel A: Logistic regression of sleep duration trajectory patterns and risk of PD, using participants without a PD diagnosis as the reference group.
Panel B: Logistic regression of sleep duration trajectory patterns and risk of "Probable pPD", using participants in the "No probable pPD" group as the reference group.
Panel C: Linear regression of sleep duration trajectory patterns and age at onset.
Model 1 was unadjusted. Model 2 adjusted for age at sleep report [for risk analyses only], sex, race, family history of PD, education, and income. Model 3 further adjusted for history of brain injury, diabetes, hypertension; REM Sleep Behavior Disorder Single-Question Screen at the time of sleep report; lifetime caffeine intake, smoking status, and lifetime physical inactivity.

Supplementary Figure 2. PPMI-Online Study Sensitivity Analysis - 2

Participants with a history of anxiety or depression excluded

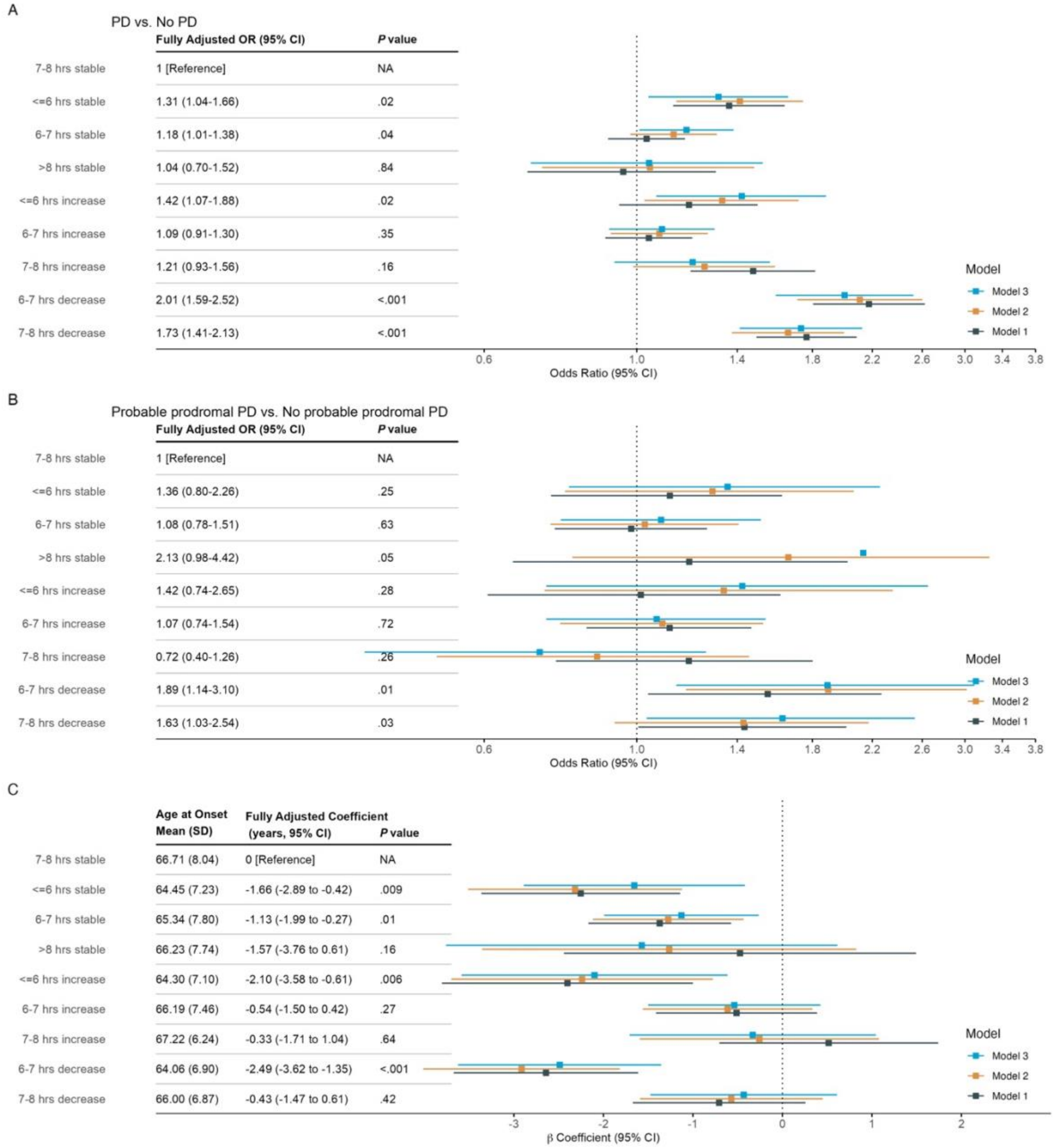

Abbreviations: OR = Odds ratio, pPD = prodromal Parkinson's disease.

Panel A: Logistic regression of sleep duration trajectory patterns and risk of PD, using participants without a PD diagnosis as the reference group.

Panel B: Logistic regression of sleep duration trajectory patterns and risk of "Probable pPD", using participants in the "No probable pPD" group as the reference group.

Panel C: Linear regression of sleep duration trajectory patterns and age at onset.

Model 1 was unadjusted. Model 2 adjusted for age at sleep report [for risk analyses only], sex, race, family history of PD, education, and income. Model 3 further adjusted for history of brain injury, diabetes, hypertension; REM Sleep Behavior Disorder Single-Question Screen at the time of sleep report; lifetime caffeine intake, smoking status, and lifetime physical inactivity.

Supplementary Figure 3. PPMI-Online Study Sensitivity Analysis - 3
Participants with possible dementia (Penn Parkinson's Daily Activities Questionnaire-15 < 43) excluded

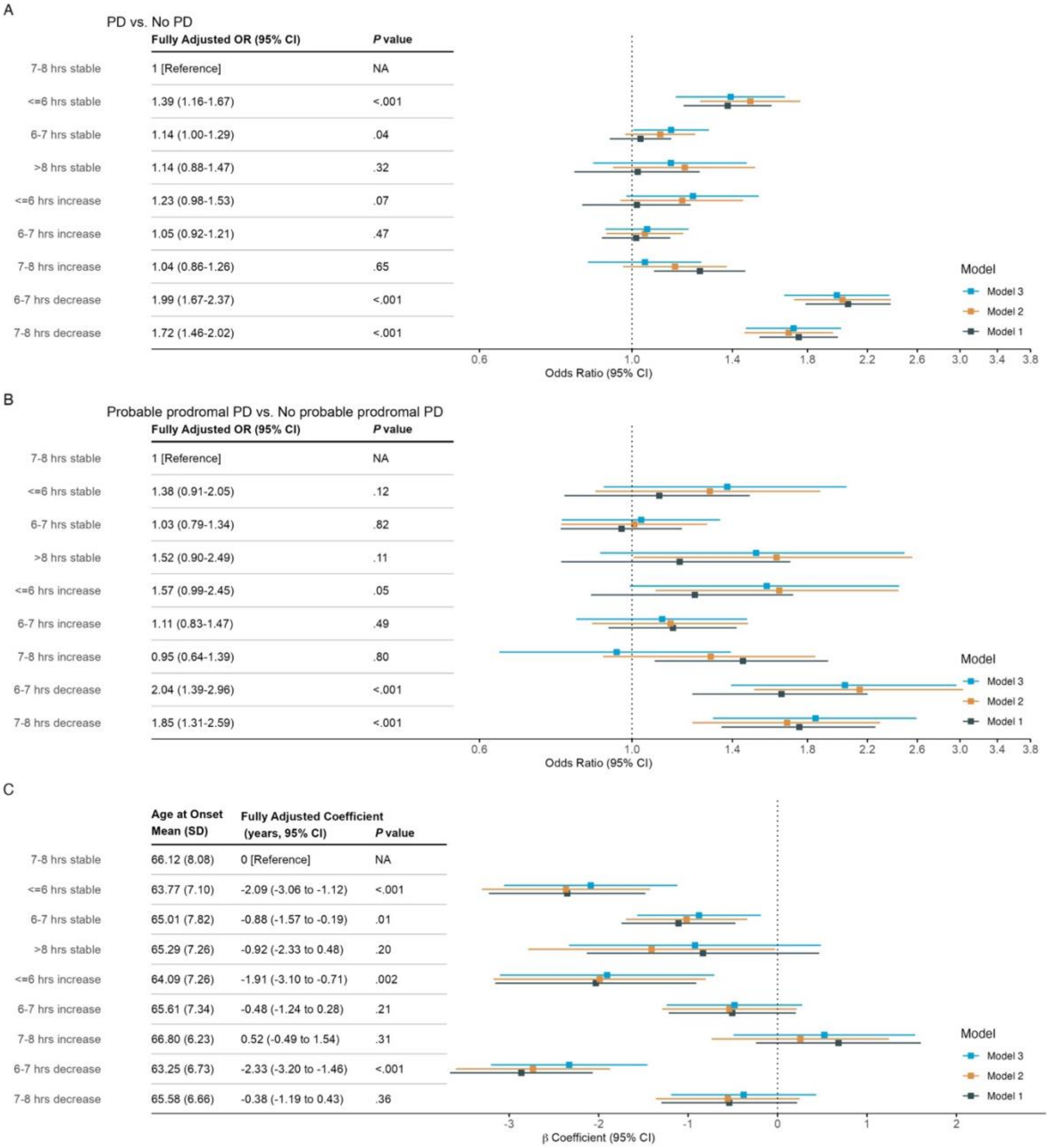

Abbreviations: OR = Odds ratio, pPD = prodromal Parkinson’s disease.
Panel A: Logistic regression of sleep duration trajectory patterns and risk of PD, using participants without a PD diagnosis as the reference group.
Panel B: Logistic regression of sleep duration trajectory patterns and risk of “Probable pPD”, using participants in the “No probable pPD” group as the reference group.
Panel C: Linear regression of sleep duration trajectory patterns and age at onset.
Model 1 was unadjusted. Model 2 adjusted for age at sleep report [for risk analyses only], sex, race, family history of PD, education, and income. Model 3 further adjusted for history of anxiety, depression, brain injury, diabetes, hypertension; REM Sleep Behavior Disorder Single-Question Screen at the time of sleep report; lifetime caffeine intake, smoking status, and lifetime physical inactivity.

Supplementary Figure 4. PPMI-Online Study Sensitivity Analysis - 4

Analysis in female

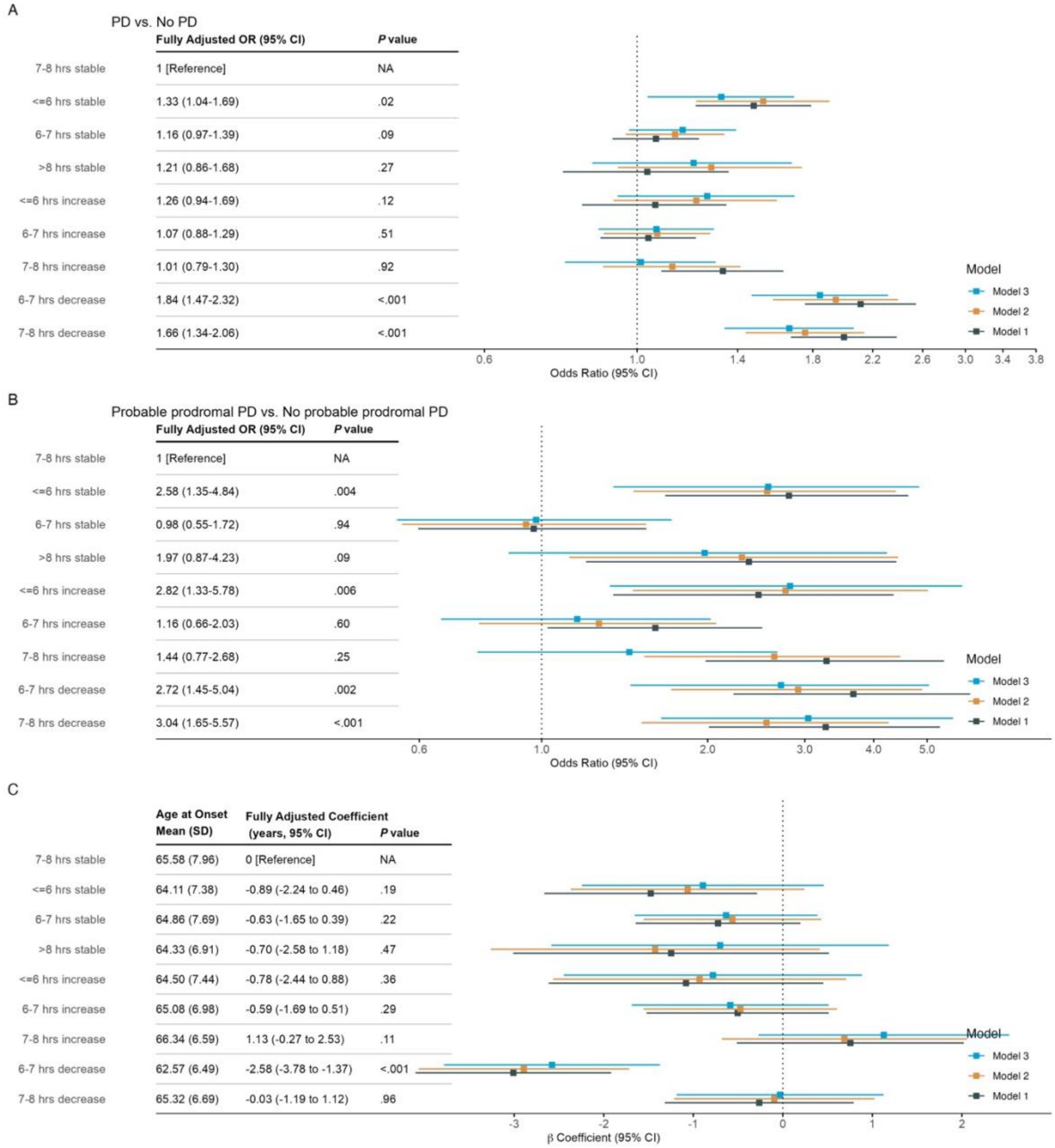

Abbreviations: OR = Odds ratio, pPD = prodromal Parkinson's disease.

Panel A: Logistic regression of sleep duration trajectory patterns and risk of PD, using participants without a PD diagnosis as the reference group.

Panel B: Logistic regression of sleep duration trajectory patterns and risk of "Probable pPD", using participants in the "No probable pPD" group as the reference group.

Panel C: Linear regression of sleep duration trajectory patterns and age at onset.

Model 1 was unadjusted. Model 2 adjusted for age at sleep report [for risk analyses only], race, family history of PD, education, and income. Model 3 further adjusted for history of anxiety, depression, brain injury, diabetes, hypertension; REM Sleep Behavior Disorder Single-Question Screen at the time of sleep report; lifetime caffeine intake, smoking status, and lifetime physical inactivity.

Analysis in male

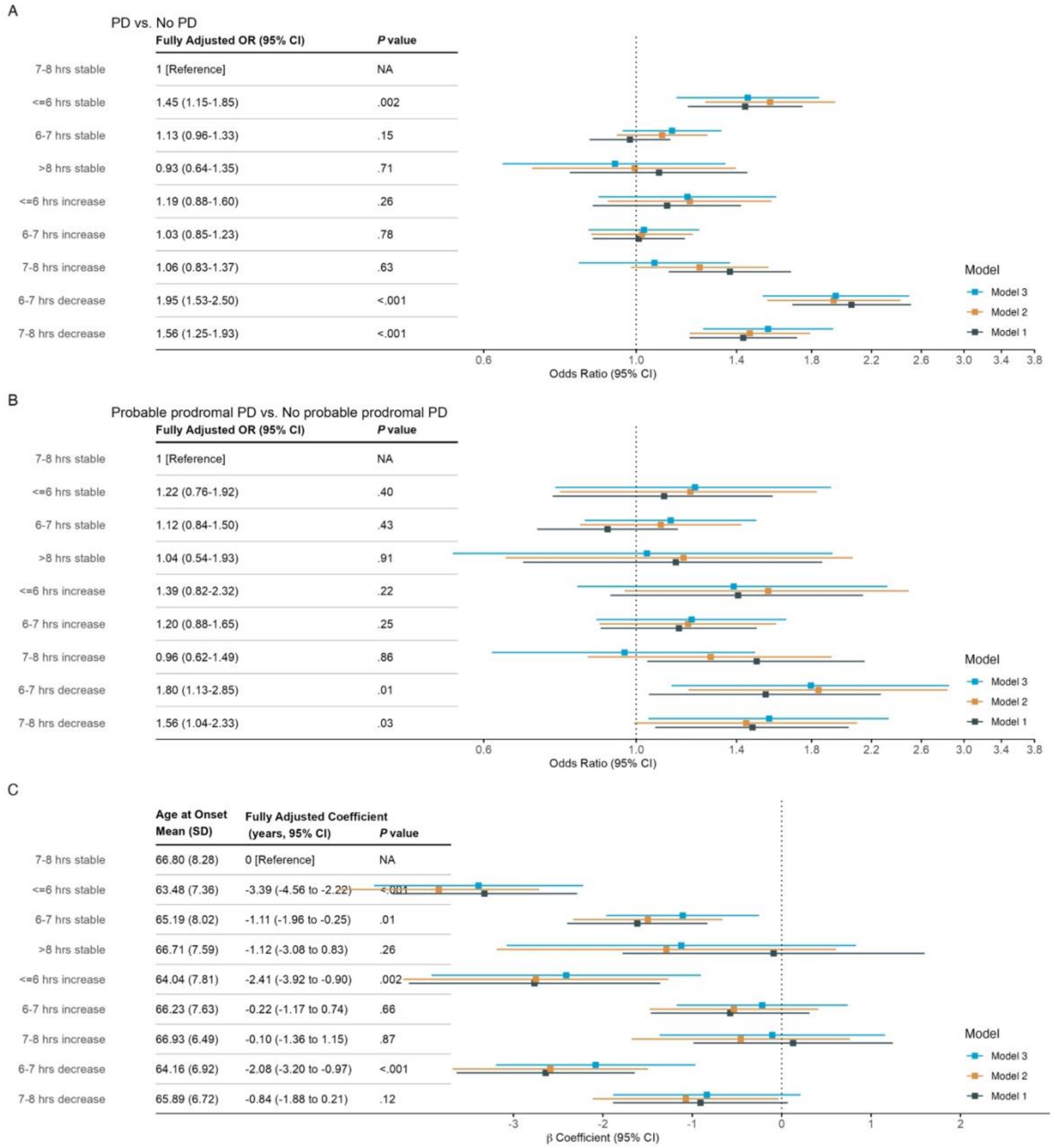

Abbreviations: OR = Odds ratio, pPD = prodromal Parkinson's disease.
Panel A: Logistic regression of sleep duration trajectory patterns and risk of PD, using participants without a PD diagnosis as the reference group.
Panel B: Logistic regression of sleep duration trajectory patterns and risk of "Probable pPD", using participants in the "No probable pPD" group as the reference group.
Panel C: Linear regression of sleep duration trajectory patterns and age at onset.
Model 1 was unadjusted. Model 2 adjusted for age at sleep report [for risk analyses only], race, family history of PD, education, and income. Model 3 further adjusted for history of anxiety, depression, brain injury, diabetes, hypertension; REM Sleep Behavior Disorder Single-Question Screen at the time of sleep report; lifetime caffeine intake, smoking status, and lifetime physical inactivity.

Supplementary Figure 5. FI Study Sensitivity Analysis - 1
Participants with a history of anxiety or depression excluded

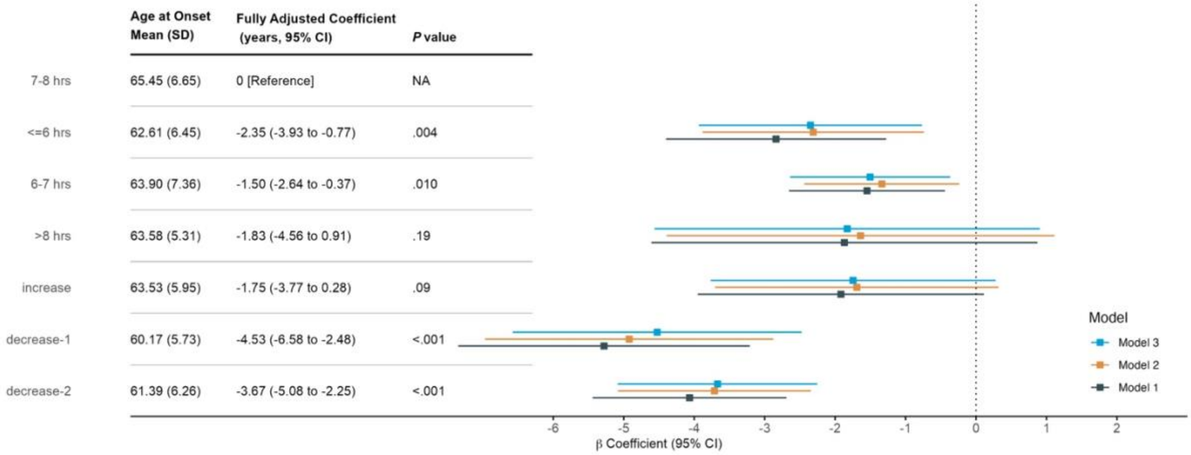

	Age at Onset Mean (SD)	Fully Adjusted Coefficient (years, 95% CI)	*P* value
7-8 hrs	65.45 (6.65)	0 [Reference]	NA
<=6 hrs	62.61 (6.45)	-2.35 (-3.93 to -0.77)	.004
6-7 hrs	63.90 (7.36)	-1.50 (-2.64 to -0.37)	.010
>8 hrs	63.58 (5.31)	-1.83 (-4.56 to 0.91)	.19
increase	63.53 (5.95)	-1.75 (-3.77 to 0.28)	.09
decrease-1	60.17 (5.73)	-4.53 (-6.58 to -2.48)	<.001
decrease-2	61.39 (6.26)	-3.67 (-5.08 to -2.25)	<.001

Model 1 was unadjusted. Model 2 adjusted for sex, race, family history of PD, education, and income. Model 3 further adjusted for history of brain injury, diabetes, hypertension; REM Sleep Behavior Disorder Single-Question Screen at the time of sleep report; lifetime caffeine intake, smoking status, and lifetime physical inactivity.

Supplementary Figure 6. FI Study Sensitivity Analysis - 2
Participants with possible dementia (Penn Parkinson's Daily Activities Questionnaire-15 < 43) excluded

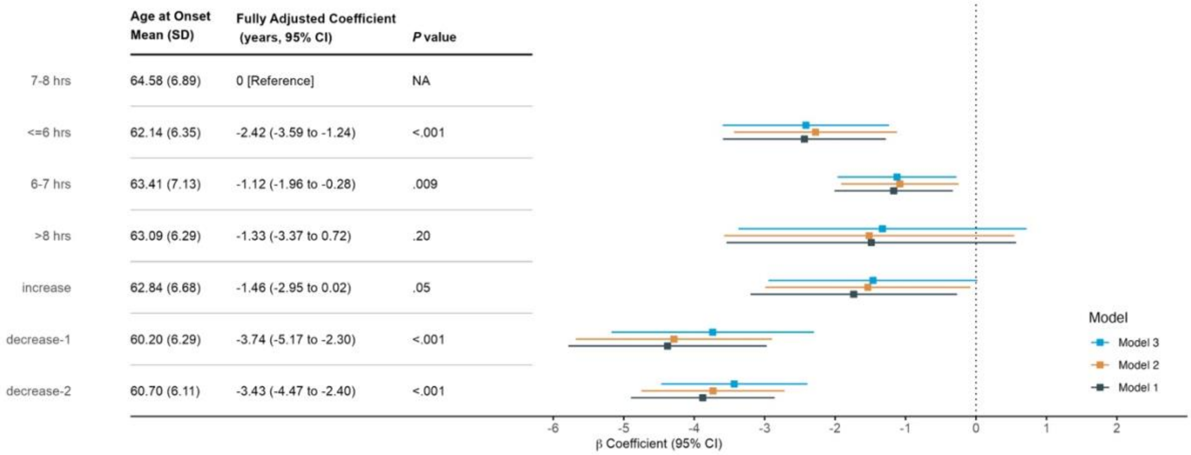

	Age at Onset Mean (SD)	Fully Adjusted Coefficient (years, 95% CI)	*P* value
7-8 hrs	64.58 (6.89)	0 [Reference]	NA
<=6 hrs	62.14 (6.35)	-2.42 (-3.59 to -1.24)	<.001
6-7 hrs	63.41 (7.13)	-1.12 (-1.96 to -0.28)	.009
>8 hrs	63.09 (6.29)	-1.33 (-3.37 to 0.72)	.20
increase	62.84 (6.68)	-1.46 (-2.95 to 0.02)	.05
decrease-1	60.20 (6.29)	-3.74 (-5.17 to -2.30)	<.001
decrease-2	60.70 (6.11)	-3.43 (-4.47 to -2.40)	<.001

Model 1 was unadjusted. Model 2 adjusted for sex, race, family history of PD, education, and income. Model 3 further adjusted for history of anxiety, depression, brain injury, diabetes, hypertension; REM Sleep Behavior Disorder Single-Question Screen at the time of sleep report; lifetime caffeine intake, smoking status, and lifetime physical inactivity.

Supplementary Figure 7. FI Study Sensitivity Analysis - 3

Analysis in female

	Age at Onset Mean (SD)	Fully Adjusted Coefficient (years, 95% CI)	*P* value
7-8 hrs	63.57 (6.63)	0 [Reference]	NA
<=6 hrs	61.63 (6.08)	-2.14 (-3.66 to -0.62)	.006
6-7 hrs	62.76 (6.96)	-0.73 (-1.83 to 0.37)	.20
>8 hrs	61.64 (5.44)	-2.03 (-4.92 to 0.87)	.17
increase	62.99 (6.55)	-0.40 (-2.23 to 1.44)	.67
decrease-1	59.47 (5.96)	-3.45 (-5.25 to -1.66)	<.001
decrease-2	60.66 (6.03)	-2.89 (-4.20 to -1.57)	<.001

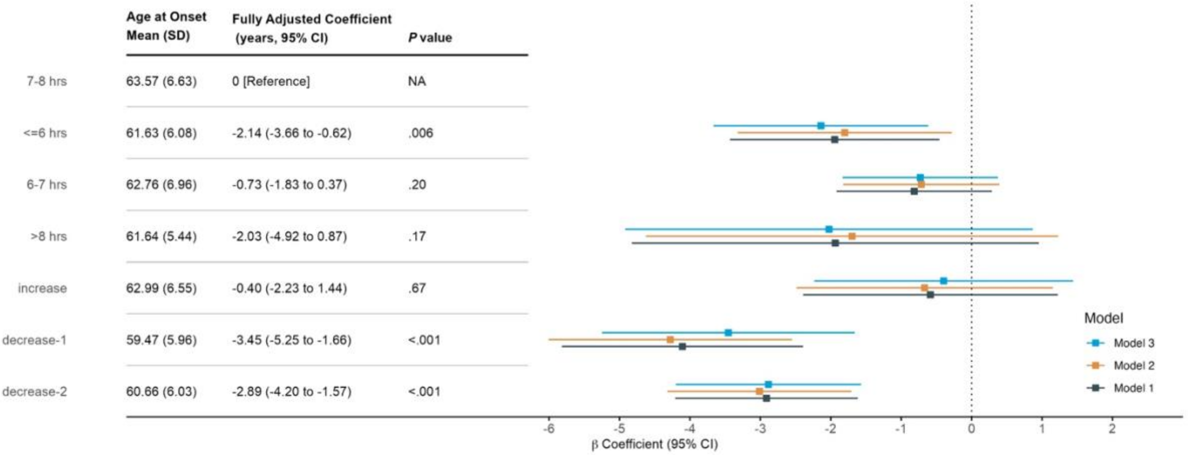

Analysis in male

	Age at Onset Mean (SD)	Fully Adjusted Coefficient (years, 95% CI)	*P* value
7-8 hrs	65.68 (7.13)	0 [Reference]	NA
<=6 hrs	62.07 (6.61)	-3.31 (-4.93 to -1.68)	<.001
6-7 hrs	63.70 (7.37)	-1.94 (-3.10 to -0.78)	.001
>8 hrs	64.32 (7.03)	-0.92 (-3.48 to 1.63)	.48
increase	62.73 (7.42)	-2.66 (-4.77 to -0.55)	.01
decrease-1	59.81 (6.42)	-5.31 (-7.21 to -3.42)	<.001
decrease-2	60.97 (6.41)	-4.09 (-5.56 to -2.63)	<.001

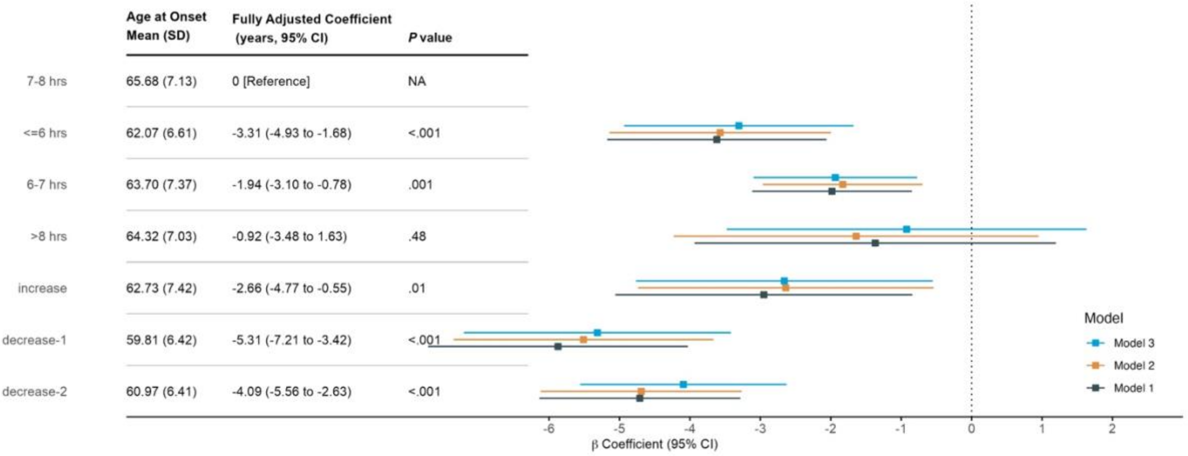

Model 1 was unadjusted. Model 2 adjusted for race, family history of PD, education, and income. Model 3 further adjusted for history of anxiety, depression, brain injury, diabetes, hypertension; REM Sleep Behavior Disorder Single-Question Screen at the time of sleep report; lifetime caffeine intake, smoking status, and lifetime physical inactivity.

Supplementary Figure 8. Risk of Parkinson's Disease and Prodromal Parkinson's Disease, and Age at Onset for Baseline and Trend Groups from Sleep Duration Trajectories.

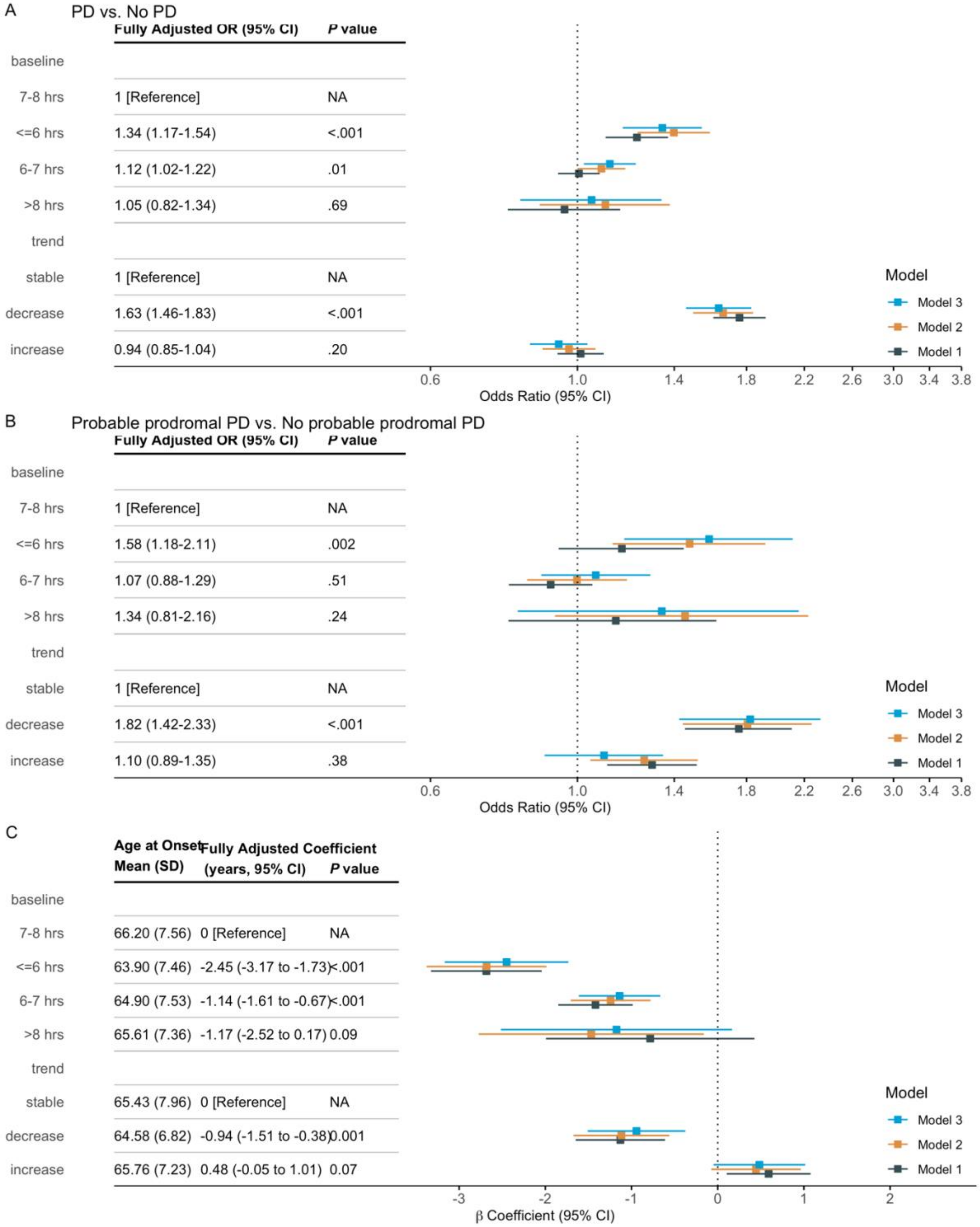

A — PD vs. No PD

	Fully Adjusted OR (95% CI)	*P* value
baseline		
7-8 hrs	1 [Reference]	NA
<=6 hrs	1.34 (1.17-1.54)	<.001
6-7 hrs	1.12 (1.02-1.22)	.01
>8 hrs	1.05 (0.82-1.34)	.69
trend		
stable	1 [Reference]	NA
decrease	1.63 (1.46-1.83)	<.001
increase	0.94 (0.85-1.04)	.20

B — Probable prodromal PD vs. No probable prodromal PD

	Fully Adjusted OR (95% CI)	*P* value
baseline		
7-8 hrs	1 [Reference]	NA
<=6 hrs	1.58 (1.18-2.11)	.002
6-7 hrs	1.07 (0.88-1.29)	.51
>8 hrs	1.34 (0.81-2.16)	.24
trend		
stable	1 [Reference]	NA
decrease	1.82 (1.42-2.33)	<.001
increase	1.10 (0.89-1.35)	.38

C

	Age at Onset Mean (SD)	Fully Adjusted Coefficient (years, 95% CI)	*P* value
baseline			
7-8 hrs	66.20 (7.56)	0 [Reference]	NA
<=6 hrs	63.90 (7.46)	-2.45 (-3.17 to -1.73)	<.001
6-7 hrs	64.90 (7.53)	-1.14 (-1.61 to -0.67)	<.001
>8 hrs	65.61 (7.36)	-1.17 (-2.52 to 0.17)	0.09
trend			
stable	65.43 (7.96)	0 [Reference]	NA
decrease	64.58 (6.82)	-0.94 (-1.51 to -0.38)	0.001
increase	65.76 (7.23)	0.48 (-0.05 to 1.01)	0.07

Abbreviations: OR = Odds ratio, pPD = prodromal Parkinson's disease.
Panel A: Logistic regression of baseline and trends in sleep duration patterns and risk of PD, using participants without a PD diagnosis as the reference group.
Panel B: Logistic regression of baseline and trends in sleep duration patterns and risk of "Probable pPD", using participants in the "No probable pPD" group as the reference group.

Panel C: Linear regression of baseline and trends in sleep duration patterns and age at onset.
Model 1 was unadjusted. Model 2 adjusted for age at sleep report [for risk analyses only], sex, race, family history of PD, education, and income. Model 3 further adjusted for history of brain injury, diabetes, hypertension; REM Sleep Behavior Disorder Single-Question Screen at the time of sleep report; lifetime caffeine intake, smoking status, and lifetime physical inactivity.

Supplementary Table 1. Demographic and Clinical Characteristics of Subgroups in PPMI-Online

	No Probable pPD (n = 5818)	Probable pPD (n = 1104)	PD (n = 5660)	*P* value[a]
Age at sleep report Mean (SD)	65.3 (7.74)	70.1 (7.50)	69.7 (7.27)	<.001, .10
Age at onset of PD Mean (SD)	NA	NA	65.3 (7.57)	NA
Follow-up since enrollment (months) Mean (SD)	23.7 (8.31)	22.3 (8.58)	22.2 (8.91)	<.001, .72
Sex (Female) No (%)	4138 (71.1%)	255 (23.1%)	2367 (41.8%)	<.001, <.001
Race (Non-White) No (%)	195 (3.5%)	28 (2.5%)	196 (3.6%)	.10, .04
Years of education Mean (SD)	17.0 (3.14)	17.1 (3.21)	16.5 (3.56)	.688, <.001
Yearly Household Income No (%)				.001, .31
<$50,000	500 (8.6%)	138 (12.5%)	707 (12.5%)	
$50,000 to $99,999	1488 (25.6%)	304 (27.5%)	1517 (26.8%)	
More than $100,000	2973 (51.1%)	517 (46.8%)	2473 (43.7%)	
Prefer not to answer	772 (13.3%)	133 (12.0%)	791 (14.0%)	
Missing	85 (1.5%)	12 (1.1%)	172 (3.0%)	
Family history of PD No (%)	3653 (62.8%)	574 (52.0%)	1664 (29.4%)	<.001, <.001
MDS-UPDRS part I Mean (SD)	4.37 (3.27)	7.14 (4.59)	8.84 (4.51)	<.001, <.001
MDS-UPDRS part II Mean (SD)	1.08 (2.15)	3.34 (4.68)	10.5 (7.49)	<.001, <.001
PDAQ-15 Mean (SD)	55.8 (4.78)	52.3 (7.68)	51.0 (9.11)	<.001, <.001
GDS-15 Mean (SD)	1.90 (2.45)	3.07 (3.27)	3.68 (3.44)	<.001, <.001
PAS Mean (SD)	6.14 (5.82)	8.22 (7.69)	9.44 (7.55)	<.001, <.001
ESS Mean (SD)	5.39 (3.27)	7.40 (3.99)	7.18 (4.20)	<.001, .10
PDSS2 Mean (SD)	10.5 (6.01)	14.0 (8.07)	16.5 (8.46)	<.001, <.001

Abbreviations: PD = Parkinson's disease, pPD = prodromal Parkinson's disease, MDS-UPDRS = Movement Disorder Society-sponsored revision of the Unified Parkinson's Disease Rating Scale, PDAQ15 = The Penn Parkinson's Daily Activities Questionnaire-15, GDS-15 = Geriatric Depression Scale-15, PAS = Parkinson Anxiety Scale, ESS = Epworth Sleepiness Scale, PDSS2 = The Parkinson's Disease Sleep Scale–2
[a]: Independent t-test for continuous variable, chi-square test for categorical variable.

Supplementary Table 2. Demographic Information of Sleep Trajectory Patterns of PD Participants in the PPMI-Online Study

	7-8 hrs stable (N=4226)	<=6 hrs stable (N=1183)	6-7 hrs stable (N=3365)	>8 hrs stable (N=509)	<=6 hrs increase (N=718)	6-7 hrs increase (N=2323)	7-8 hrs increase (N=1039)	6-7 decrease (N=1162)	7-8 hrs decrease (N=1380)	*P* value
Age at sleep report Mean (SD)	67.1 (8.78)	65.8 (7.66)***	66.0 (8.59)***	66.0 (8.05)	66.4 (7.76)	67.5 (7.32)	69.8 (6.07)***	68.1 (5.98)***	69.5 (6.38)***	<.001
Female n (%)	2315 (54.8%)	645 (54.5%)	1808 (53.7%)	315 (61.9%)*	428 (59.6%)	1311 (56.4%)	582 (56.0%)	647 (55.7%)	744 (53.9%)	.01
Race (White)		***			*					<.001
No	78 (1.8%)	72 (6.1%)	84 (2.5%)	7 (1.4%)	27 (3.8%)	44 (1.9%)	9 (0.9%)	38 (3.3%)	30 (2.2%)	
Yes	4101 (97.0%)	1084 (91.6%)	3231 (96.0%)	497 (97.6%)	679 (94.6%)	2260 (97.3%)	1019 (98.1%)	1108 (95.4%)	1331 (96.4%)	
Unknown or PNA	16 (0.4%)	11 (0.9%)	17 (0.5%)	1 (0.2%)	5 (0.7%)	5 (0.2%)	2 (0.2%)	4 (0.3%)	5 (0.4%)	
Missing	31 (0.7%)	16 (1.4%)	33 (1.0%)	4 (0.8%)	7 (1.0%)	14 (0.6%)	9 (0.9%)	12 (1.0%)	14 (1.0%)	
Years of Education	16.9 (3.50)	16.3 (3.44)***	16.8 (3.34)	17.1 (3.45)	16.8 (3.07)	17.0 (3.35)	16.9 (2.94)	16.4 (3.29)***	16.6 (3.07)	<.001
Yearly Household Income		*					*	***	***	<.001
<$20,000	55 (1.3%)	36 (3.0%)	44 (1.3%)	15 (2.9%)	16 (2.2%)	23 (1.0%)	19 (1.8%)	33 (2.8%)	25 (1.8%)	
$20,000 to $34,999	132 (3.1%)	73 (6.2%)	100 (3.0%)	23 (4.5%)	27 (3.8%)	71 (3.1%)	47 (4.5%)	76 (6.5%)	72 (5.2%)	
$35,000 to $49,999	233 (5.5%)	72 (6.1%)	153 (4.5%)	28 (5.5%)	33 (4.6%)	125 (5.4%)	56 (5.4%)	65 (5.6%)	84 (6.1%)	
$50,000 to $74,999	444 (10.5%)	146 (12.3%)	392 (11.6%)	68 (13.4%)	86 (12.0%)	285 (12.3%)	132 (12.7%)	172 (14.8%)	200 (14.5%)	
$75,000 to $99,999	595 (14.1%)	182 (15.4%)	437 (13.0%)	67 (13.2%)	111 (15.5%)	302 (13.0%)	164 (15.8%)	173 (14.9%)	198 (14.3%)	
More than $100,000	2114 (50.0%)	461 (39.0%)	1689 (50.2%)	232 (45.6%)	332 (46.2%)	1155 (49.7%)	442 (42.5%)	448 (38.6%)	571 (41.4%)	
Prefer Not to Answer	545 (12.9%)	170 (14.4%)	444 (13.2%)	64 (12.6%)	96 (13.4%)	302 (13.0%)	141 (13.6%)	162 (13.9%)	198 (14.3%)	
Missing	108 (2.6%)	43 (3.6%)	106 (3.2%)	12 (2.4%)	17 (2.4%)	60 (2.6%)	38 (3.7%)	33 (2.8%)	32 (2.3%)	

[a] ANOVA was used for continuous variable, and the chi-square test was used for categorical variable

*: represent significant difference from the reference group ("7-8 hrs stable") in the post hoc test

*: $P < 0.05$

**: $P < 0.01$

***: $P < 0.001$

Supplementary Table 3. Demographic Information of Sleep Trajectory Patterns of PD Participants in the Fox Insight Study

	7-8 hrs<br>n = 563	<=6 hrs<br>n = 208	6-7 hrs<br>n = 565	>8 hrs<br>n = 53	decrease-1<br>n = 139	decrease-2<br>n = 289	increase<br>n = 112	*P* value [a]
Age at sleep report	68.9 (6.87)	66.8 (6.36)	67.8 (6.97)	67.7 (6.52)	64.2 (6.36) ***	65.9 (6.47) ***	67.6 (6.87)	<.001
Sex (female)	271 (48.1%)	101 (48.6%)	265 (46.9%)	21 (39.6%)	70 (50.4%)	151 (52.2%)	61 (54.5%)	.43
Race (White)	555 (98.6%)	203 (97.6%)	550 (97.3%)	51 (96.2%)	136 (97.8%)	286 (99.0%)	110 (98.2%)	.53
Education		***	*		**	***		<.001
<Bachelor's	138 (24.5%)	94 (45.2%)	170 (30.1%)	14 (26.4%)	52 (37.4%)	106 (36.7%)	33 (29.5%)	
>=Bachelor's	425 (75.5%)	112 (53.8%)	394 (69.7%)	39 (73.6%)	87 (62.6%)	181 (62.6%)	78 (69.6%)	
Yearly Household Income								.22
<$50,000	117 (20.8%)	63 (30.3%)	119 (21.1%)	15 (28.3%)	35 (25.2%)	67 (23.2%)	36 (32.1%)	
$50,000 to $74,999	110 (19.5%)	36 (17.3%)	95 (16.8%)	10 (18.9%)	19 (13.7%)	60 (20.8%)	16 (14.3%)	
$75,000 and above	253 (44.9%)	84 (40.4%)	260 (46.0%)	21 (39.6%)	60 (43.2%)	117 (40.5%)	46 (41.1%)	

[a] ANOVA was used for continuous variable, and the chi-square test was used for categorical variable

*: represent significant difference from the reference group ("7-8 hrs") in the post hoc test

*: $P < 0.05$

**: $P < 0.01$

***: $P < 0.001$
